# Polyoxygenated Cembrane Diterpenoids from the Soft Coral *Sarcophyton ehrenbergi*

**DOI:** 10.3390/ijms16036140

**Published:** 2015-03-17

**Authors:** Shi-Yie Cheng, Shang-Kwei Wang, Mu-Keng Hsieh, Chang-Yih Duh

**Affiliations:** 1 Department of Life Sciences, National University of Kaohsiung, Kaohsiung 811, Taiwan; E-Mail: shiyie@nuk.edu.tw; 2 Department of Microbiology, Kaohsiung Medical University, Kaohsiung 807, Taiwan; E-Mail: skwang@cc.kmu.edu.tw; 3 Department of Marine Biotechnology and Resources, National Sun Yat-sen University, Kaohsiung 804, Taiwan; E-Mail: qaz7824780@yahoo.com.tw; 4 Graduate Institute of Natural Products, Kaohsiung Medical University, Kaohsiung 807, Taiwan

**Keywords:** polyoxygenated cembranoids, *Sarcophyton ehrenbergi*, cytotoxicity, HCMV (human cytomegalovirus)

## Abstract

Five new polyoxygenated cembranoids, named (+)-1,15-epoxy-2-methoxy-12-methoxycarbonyl-11*E*-sarcophytoxide (**1**), (+)-2-*epi*-12-methoxycarbonyl-11*E*-sarcophine (**2**), 3,4-epoxyehrenberoxide A (**3**), ehrenbergol D (**4**) and ehrenbergol E (**5**), were obtained from the soft coral *Sarcophyton ehrenbergi*. The structures of **1**–**5** were established on the basis of comprehensive NMR and HR-ESI-MS analyses and by comparison with reported data in the literature. Compounds **4** and **5** showed moderate cytotoxicity against P-388 (mouse lymphocytic leukemia) cancer cell line with EC_50_ values of 2.0 and 3.0 μM, respectively. Compound **2** exhibited slight antiviral activity against HCMV (human cytomegalovirus) with IC_50_ values of 25.0 μg/mL.

## 1. Introduction

*Sarcophyton ehrenbergi* (von Marenzeller, 1886), ordinarily regarded as the leather coral, belongs to the order Alcyonacea, and is classified as an octocoral because of eight-fold symmetry or eight-branched tentacles in their polyp structure. From a chemical perspective, the alcyonacean soft coral *S. ehrenbergi* is interesting due to macrocyclic cembranoids with intriguing structural features and various promising biological activities [1,2,3,4,5,6,7]. In the course of our ongoing research focused toward the purification of bioactive metabolites from the organism, several polyoxygenated cembrane-type diterpenoids were discovered. Some of the obtained metabolites have been proven to exhibit antiviral and cytotoxic properties [4,5,6]. The two soft corals form our previous studies on the bioactive metabolites of *S. ehrenbergi* were collected at the Dongsha atoll and San-Hsian-Tai island, respectively. The comparative results revealed that the two samples collected from the different habitat might have different secondary metabolites even though they are supposed to be the same species. Apparently, some of these obtained cembranoids are able to be produced or modified through their symbiosis with zooxanthellae [8].

**Figure 1:**
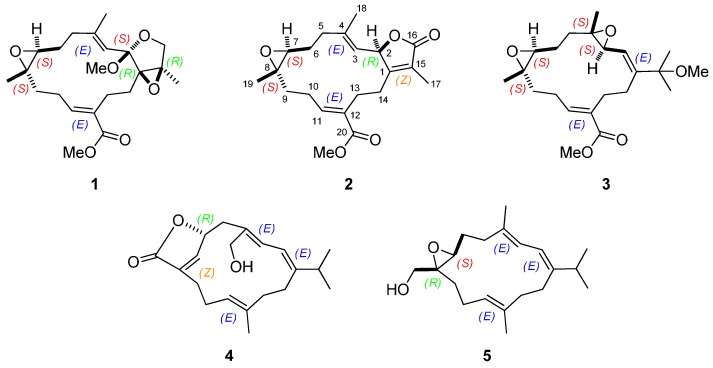
The structures of (+)-1,15-epoxy-2-methoxy-12-methoxycarbonyl-11*E*-sarcophytoxide (**1**), (+)-2-*epi*-12-methoxycarbonyl-11*E*-sarcophine (**2**), 3,4-epoxyehrenberoxide A (**3**), ehrenbergol D (**4**) and ehrenbergol E (**5**).

In view of the bioactive potential of macrocyclic cembrane-type diterpenoids, our continuing chemical investigations of *S. ehrenbergi*, collected at San-Hsian-Tai island (Taitong County), have resulted in the purification of five new polyoxygenated cembranoids **1**–**5** (Figure 1) from the acetone-soluble fraction of the organism. The structures of these obtained metabolites were elucidated through extensive spectroscopic analyses, including 2D NMR spectroscopy [correlation spectroscopy (COSY), heteronuclear single-quantum correlation (HSQC), heteronuclear multiple bond coherence (HMBC) and nuclear overhauser effect spectroscopy (NOESY)], and by comparison of the spectroscopic data with those of related known compounds [4,5,6,7]. Moreover, compounds **1**–**5** were evaluated *in vitro* for cytotoxicity against P-388 (mouse lymphocytic leukemia), A-459 (human lung carcinoma), HT-29 (human colon adenocarcinoma), and HEL (human embryonic lung) cells, and antiviral activity against HCMV (human cytomegalovirus).

## 2. Results and Discussion

**Figure 2:**
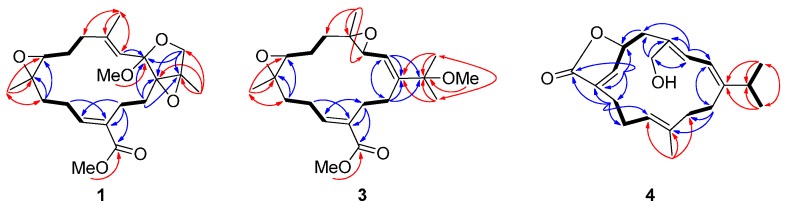
Selected ^1^H−^1^H COSY (▬) and HMBC (→) correlations of **1**, **3** and **4**.

Specimens of *S. ehrenbergi* were stored in a freezer until extraction. Conventional extraction procedures were used, and the acetone extract was exhaustively partitioned between EtOAc and H_2_O to afford the EtOAc-soluble fraction, which was evaporated under vacuum to yield a dark brown gum (46.9 g). The concentrated residue was subjected to column chromatography and high-performance liquid chromatography, leading to the purification of compounds **1**–**5**.

(+)-1,15-Epoxy-2-methoxy-12-methoxycarbonyl-11*E*-sarcophytoxide (**1**) had a high resolution electrospray ionization mass spectrometry (HRESIMS) sodiated molecular ion peak at *m*/*z* 415.2093 [M + Na]^+^, corresponding to the molecular formula C_22_H_32_O_6_ with seven indices of hydrogen deficiency. The IR spectrum of **1** showed a strong absorption band at 1712 cm^−1^, indicating the presence of an α,β-unsaturated methyl ester carbonyl group, as well as from the UV absorption (MeOH) λ_max_ (log ε) at 225 (3.83) nm [4]. This functionality was further identified by the ^1^H NMR signals (Table 1) at δ_H_ 6.85 (1H, dd, *J* = 10.0, 6.8 Hz) and ^13^C NMR signals (Table 2) at δ_C_ 167.4 (qC, C-20), 133.8 (qC, C-12) and 141.4 (CH, C-11). Comparison of the NMR spectroscopic data (Table 1 and Table 2) coupled with ^1^H–^1^H COSY, HSQC and HMBC correlations (Figure 2) of **1** with those of lobophynin C [4], suggested that **1** was established to be an 1,15-epoxy-2-methoxylated analogue of lobophynin C, coinciding with methoxy protons at δH 3.18 (3H, s) correlated to a ketal carbon resonating at δ_C_ 107.1 (qC, C-2), and methyl protons at δH 1.11 (3H, s, Me-17) correlated to the quaternary oxycarbons at δC 71.7 (qC, C-1) and 64.6 (qC, C-15) in its HMBC spectrum.

**Figure 3:**
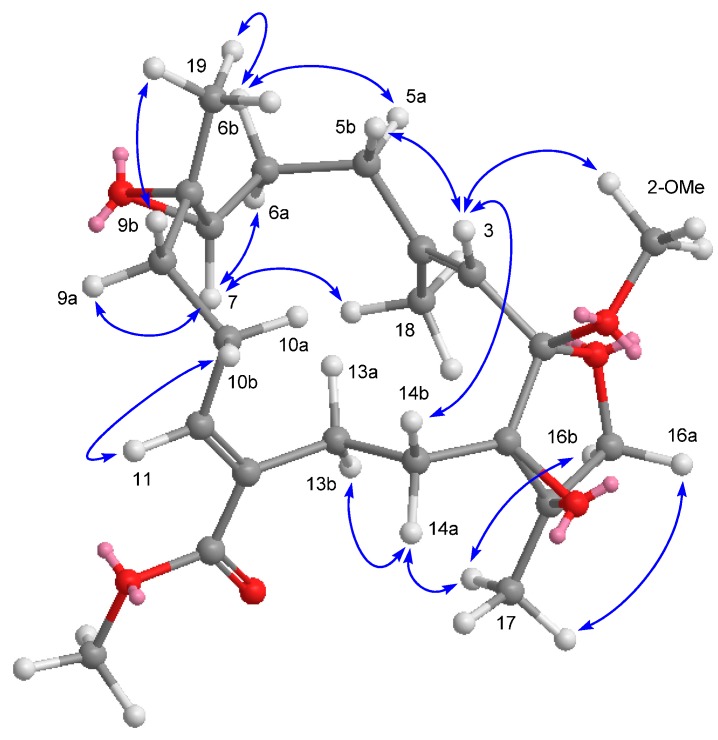
Selected NOESY correlations and computer-generated perspective model using MM2 force field calculations for **1**.

The relative stereochemistry of **1** was facilitated by the NOESY correlations and previously reported X-ray crystallographic data of (+)-12-carboxy-11*E*-sarcophytoxide [4]. The absence of a NOESY correlation (Figure 3) between the vinylic H-11 and H_2_-13 made it possible to identify the configuration of the olefin at C-11/C-12 as *E* geometry, which was also confirmed by the chemical shift of H-11 at δ_H_ 6.85 [9]. The geometry of the trisubstituted olefin at C-3 and C-4 was assigned as *E* based on the γ-effect of the olefinic methyl signal for CH_3_-18 (<20 ppm) [10]. Moreover, the crucial NOESY correlations (Figure 3) between H-3/2-OMe, H-3/H-5b (δ_H_ 1.95), H-3/H-14b (δ_H_ 1.96), H-7/Me-18, H-7/H-9a (δ_H_ 1.85), H-7/H-6a (δ_H_ 2.00), Me-19/H-9b (δ_H_ 0.96), Me-19/H-6b (δ_H_ 1.22), H-11/H-10b (δ_H_ 1.79), H-13b (δ_H_ 2.49)/H-14a (δ_H_ 2.25), and H-14a/Me-17 demonstrated the 1R*, 2S*, 7S*, 8S* and 15R* configurations. By correlation with ehrenberoxides on the biogenetic consideration, the absolute configurations of the key chiral carbons in **1** could be proposed as depicted in Figure 1. Our result presented here has added further support to this conclusion that almost all cembrane diterpenes of known absolute configuration at C-1 reported from the order Alcyonacea possess the *R*-configuration [11,12].

The HRESIMS spectrum exhibited a sodiated molecular ion peak at *m*/*z* 383.1832 [M + Na]^+^ for **2** identical to that of (+)-12-methoxycarbonyl-11*E*-sarcophine [4]. Comprehensive analysis of 2D NMR data, comprising COSY, HMQC and HMBC experiments, enabled the complete planar structure of **2**, the same as that of lobophynin C. The crucial NOESY correlations between H-7/H-6a (δ_H_ 2.21), Me-19/H-6b (δ_H_ 1.47), H-7/H-9a (δ_H_ 2.19), and Me-19/H-9b (δ_H_ 1.08) indicated the same disposition of the epoxide at C-7 and C-8 as in the case of **1**. Owing to the remarkable chemical shift changes of H-2, H-3 and H_3_-18 (δ_H_ 5.57, 5.08, and 1.87, respectively in CDCl_3_ of (+)-12-methoxycarbonyl-11*E*-sarcophine), we assume that the only variety between **2** and (+)-12-methoxycarbonyl-11*E*-sarcophine is the different orientation of the α,β-unsaturated γ-lactone ring fused to the 14-membered ring at C-2. The CD spectrum of **2** exhibited a positive Cotton effect around λ_max_ (Δε) 279 (+6.8) nm and a negative Cotton effect around λ_max_ (Δε) 245 (−1.1) and 215 (−2.9) nm, suggesting the *R*-configuration at C-2 [13]. Hence, compound **2** was assigned as (+)-2-*epi*-12-methoxycarbonyl-11*E*-sarcophine.

**Table 1:** ^1^H NMR spectroscopic data of compounds **1**–**5**.

No.	1 *^a^*	2 *^b^*	3 *^a^*	4 *^a^*	5 *^b^*
2	-	5.48 d (9.2) *^c^*	5.43 d (8.4) *^c^*	5.89 d (11.2) *^c^*	6.07 d (10.8) *^c^*
3	5.33 s	5.22 d (9.2)	3.53 d (8.4)	6.29 d (11.2)	5.98 d (10.8)
5	a: 2.03 m	a: 2.54 m	a: 2.02 ddd (14.0, 5.6, 2.8)	a: 2.87 m	a: 2.28 m
	b: 1.95 m	b: 2.19 m	b: 1.08 m	b: 2.83 m	b: 2.24 m
6	a: 2.00 m	a: 2.21 m	a: 1.83 m	5.19 m	a: 1.85 m
	b: 1.22 m	b: 1.47 m	b: 1.16 m	-	b: 1.79 m
7	2.74 dd (10.0, 3.6) *^c^*	2.57 m	2.74 dd (10.8, 3.6)	6.83 br s	3.01 dd (6.0, 5.6)
9	a: 1.85 m	a: 2.19 m	a: 1.91 m	a: 2.52 m	a: 2.18 m
	b: 0.96 dd (16.4, 9.6)	b: 1.08 m	b: 0.80 t (12.4)	b: 2.04 m	b: 1.61 m
10	a: 2.09 m	a: 2.59 m	a: 1.84 m	a: 2.31 m	a: 2.18 m
	b: 1.79 m	b: 2.17 m	b: 1.65 tt (12.4, 6.0)	b: 2.18 m	b: 2.04 m
11	6.85 dd (10.0, 6.8)	6.80 dd (8.8, 6.4)	6.83 dd (10.4, 6.4)	4.66 br d (10.4)	5.08 br t (6.4)
13	a: 2.64 td (13.6, 6.4)	a: 2.55 m	a: 2.56 td (12.8, 4.8)	a: 2.22 m	2.04 m
	b: 2.49 td (13.6, 3.6)	b: 2.37 m	b: 2.40 td (12.8, 4.8)	b: 2.07 m	-
14	a: 2.25 td (13.6, 6.4)	2.19 m	a: 2.28 td (12.8, 4.8)	a: 2.68 m	2.29 m
	b: 1.96 m	-	b: 2.19 td (12.8, 4.8)	b: 1.98 m	-
15	-	-	-	-	2.35 m
16	a: 3.68 d (10.0)	-	1.32 s	1.05 d (6.8)	1.06 d (6.4)
	b: 3.38 d (10.0)	-	-	-	-
17	1.11 s	1.92 s	1.39 s	1.14 d (6.8)	1.07 d (6.4)
18	2.00 s	1.96 s	1.12 s	a: 4.09 d (13.6)	1.76 s
	-	-	-	b: 4.03 d (13.6)	-
19	1.04 s	1.31 s	0.93 s	-	a: 3.77 dd (12.0, 6.4)
	-	-	-	-	b: 3.56 dd (12.0, 4.8)
20	-	-	-	1.51 s	1.59 s
2-OMe	3.18 s	-	-	-	-
15-OMe		-	2.93 s	-	-
20-OMe	3.40 s	3.77 s	3.40 s	-	-
19-OH	-	-	-	-	1.62 m

*^a^* Spectra were measured in C_6_D_6_ (400 MHz); *^b^* Spectra were measured in CDCl_3_ (400 MHz); *^c^* *J* values (in Hz) are in parentheses.

**Table 2:** ^13^C NMR spectroscopic data of compounds **1**–**5**.

No.	1 *^a^*	2 *^b^*	3 *^a^*	4 *^b^*	5 *^b^*
1	71.7 (qC) *^c^*	161.1 (qC) *^c^*	148.8 (qC) *^c^*	149.3 (qC) *^c^*	148.2 (qC) *^c^*
2	107.1 (qC)	78.7 (CH)	123.7 (CH)	118.5 (CH)	118.3 (CH)
3	121.9 (CH)	119.3 (CH)	60.8 (CH)	126.1 (CH)	121.7 (CH)
4	144.2 (qC)	146.9 (qC)	61.4 (qC)	130.5 (qC)	133.7 (qC)
5	38.6 (CH_2_)	36.8 (CH_2_)	36.7 (CH_2_)	29.7 (CH_2_)	35.7 (CH_2_)
6	25.2 (CH_2_)	24.1 (CH_2_)	25.3 (CH_2_)	81.0 (CH)	24.9 (CH_2_)
7	61.0 (CH)	60.9 (CH)	61.3 (CH)	148.0 (CH)	61.8 (CH)
8	60.2 (qC)	60.6 (qC)	60.9 (qC)	132.8 (qC)	62.9 (qC)
9	39.2 (CH_2_)	37.6 (CH_2_)	40.1 (CH_2_)	25.9 (CH_2_)	32.2 (CH_2_)
10	25.8 (CH_2_)	26.6 (CH_2_)	26.9 (CH_2_)	26.2 (CH_2_)	39.1 (CH_2_)
11	141.4 (CH)	142.7 (CH)	141.3 (CH)	125.9 (CH)	125.6 (CH)
12	133.8 (qC)	130.7 (qC)	133.1 (qC)	133.8 (qC)	135.9 (qC)
13	23.2 (CH_2_)	24.7 (CH_2_)	28.2 (CH_2_)	38.2 (CH_2_)	22.0 (CH_2_)
14	24.2 (CH_2_)	25.9 (CH_2_)	27.6 (CH_2_)	27.6 (CH_2_)	28.4 (CH_2_)
15	64.6 (qC)	124.5 (qC)	77.8 (qC)	33.0 (CH)	34.7 (CH)
16	68.9 (CH_2_)	174.6 (qC)	25.4 (CH_3_)	23.4 (CH_3_)	22.4 (CH_3_)
17	11.6 (CH_3_)	9.1 (CH_3_)	25.5 (CH_3_)	21.5 (CH_3_)	22.1 (CH_3_)
18	16.0 (CH_3_)	15.4 (CH_3_)	16.4 (CH_3_)	68.5 (CH_2_)	17.0 (CH_3_)
19	16.1 (CH_3_)	16.3 (CH_3_)	15.3 (CH_3_)	173.6 (qC)	63.1 (CH_2_)
20	167.4 (qC)	167.5 (qC)	167.4 (qC)	16.3 (CH_3_)	17.2 (CH_3_)
2-OMe	49.2 (CH_3_)	-	-	-	-
15-OMe	-	-	49.9 (CH_3_)	-	-
20-OMe	51.2 (CH_3_)	51.9 (CH_3_)	51.3 (CH_3_)	-	-

*^a^* Spectra were measured in C_6_D_6_ (100 MHz); *^b^* Spectra were measured in CDCl_3_ (100 MHz); *^c^* Multiplicities are deduced by HSQC and DEPT experiments.

**Figure 4:**
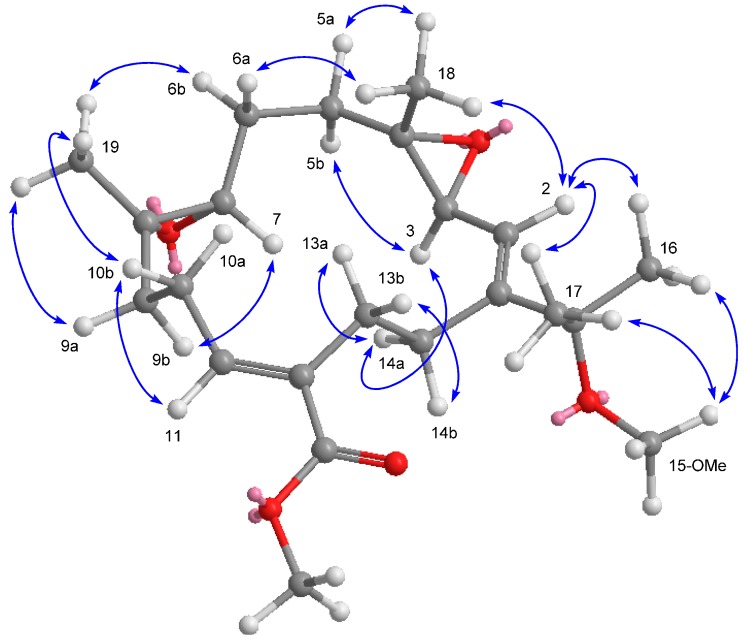
Selected NOESY correlations and computer-generated perspective model using MM2 force field calculations for **3**.

The HRESIMS of compound **3** showed a sodiated molecular ion peak at *m*/*z* 401.2301 [M + Na]^+^, which coupled with the ^13^C NMR data (Table 2), suggested a molecular formula C_22_H_34_O_5_ with six indices of hydrogen deficiency. The complete analysis of ^1^H–^1^H COSY, HMBC, and HSQC spectra permitted us to assign all the spectroscopic signals and to propose the planar structure for **3** (Figure 2). The NMR data (Table 1 and Table 2) of **3** were analogous to those of ehrenberoxide A [4] with the exception that the resonances due to the double bond at C-3 and C-4 [δ_H_ 6.10 (1H, d, *J* = 10.6 Hz) and δ_C_ 124.3 (CH) and 136.7 (qC)] in ehrenberoxide A were replaced by those due to a epoxide [δ_H_ 3.53 (1H, d, *J* = 8.4 Hz); δ_C_ 60.8 (CH) and 61.4 (qC)] in **3**. The HMBC correlations (Figure 2) from Me-18 to C-3, C-4 and C-5 further indicated that the position of the epoxide group at C-3 and C-4. Moreover, the relative configuration of epoxide was assumed to be 3S* and 4S* according to the crucial NOESY correlations between H-2/Me-18, Me-18/H-5a (δ_H_ 2.02), Me-18/H-6a (δ_H_ 1.83), H-3/H-5b (δ_H_ 1.08), and H-3/H-14a (δ_H_ 2.28) (Figure 4). The 7*S*,8*S* configuration of epoxide was concluded, consistent with previously reported (+)-12-carboxy-11*E*-sarcophytoxide for which X-ray crystallography established the absolute configuration [4]. Accordingly, compound **3** was determined unambiguously and defined as 3,4-epoxyehrenberoxide A.

**Figure 5:**
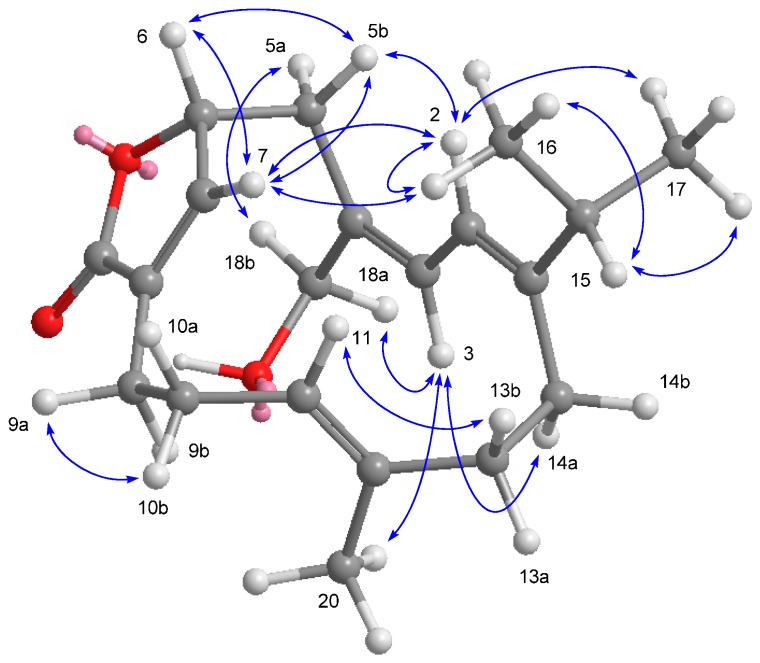
Selected NOESY correlations and computer-generated perspective model using MM2 force field calculations for **4**.

Ehrenbergol D (**4**) analyzed for the molecular formula C_20_H_28_O_3_ from its HRESIMS (*m*/*z* 339.1938, [M + Na]^+^) and NMR data (Table 1 and Table 2), suggesting seven indices of hydrogen deficiency. The IR spectrum of **4** at 3422 cm^−1^ demonstrated a broad absorption band diagnostic of a primary hydroxyl, which was further identified by the ^1^H NMR signals at δ_H_ 4.09 (1H, d, *J* = 13.6 Hz, H-18a) and 4.03 (1H, d, *J* = 13.6 Hz, H-18b), as well as the ^13^C NMR signal at δ_C_ 68.5 (CH_2_, C-18). The NMR data indicated that **4** possesses four trisubstituted double bonds [δ_H_ 5.89 (1H, d, *J* = 11.2 Hz, H-2); δ_C_ 149.3 (qC, C-1) and 118.5 (CH, C-2); δ_H_ 6.29 (1H, d, *J* = 11.2 Hz, H-3); δ_C_ 126.1 (CH, C-3) and 130.5 (qC, C-4); δ_H_ 6.83 (1H, br s, H-7); δ_C_ 148.0 (CH, C-7) and 132.8 (qC, C-8); δ_H_ 4.66 (1H, br d, *J* = 10.4 Hz, H-11); δ_C_ 125.9 (CH, C-11) and 133.8 (qC, C-12)]. Additionally, the carbon resonance appearing at δ_C_ 173.6 (qC, C-19) was attributable to an α,β-unsaturated γ-lactone carbon. Correlations deduced from extensive analysis of the ^1^H–^1^H COSY correlations of **4** enabled initially the establishment of five partial structures. The connectivity of the above structural fragments was subsequently interconnected by the HMBC correlations (Figure 2). The crucial NOESY correlations between H-2/H-5b (δ_H_ 2.83), H-2/Me-16, H-2/Me-17, H-3/Me-20, H-5a (δ_H_ 2.87)/H-18b (δ_H_ 4.03), H-3/H-18a (δ_H_ 4.09) and H-3/H-14a (δ_H_ 2.68) (Figure 5) indicated that the geometries of the conjugated diene at C-1/C-2 and C-3/C-4 were assigned as *E* and *E*, respectively. The absence of NOESY correlation between H-2 and H-3 and the large coupling constant (*J*_2,3_ = 11.2 Hz) further suggested the *s-trans* geometry of the conjugated double bonds [4]. The key NOESY correlations between H-11 and H-13b (δ_H_ 2.07) as well as the *γ*-effect of the olefinic methyl signal for CH_3_-20 (<20 ppm) [10] reflected the *E* geometry of the trisubstituted double bond at C-11 and C-12 in the molecule. The CD spectrum of **4** exhibited a negative Cotton effect around λ_max_ (Δε) 205 (−9.0) nm and a positive Cotton effect around λ_max_ (Δε) 259 (+24.9) nm, which matches with the *R* configuration of model α,β-unsaturated γ-lactone [13]. Consequently, ehrenbergol D (**4**) was designated as (1*E*,3*E*,6*R*,7*Z*,11*E*)-18-hydroxycembra-1,3,7,11-tetraen-6,19-olide.

Ehrenbergol E (**5**) was assigned a molecular formula C_20_H_32_O_2_, according to its HRESIMS and NMR spectroscopic data (Table 1 and Table 2). The decided assignment of **5** was established by the interpretation of ^1^H−^1^H COSY and HMBC correlations. The ^1^H−^13^C long-range correlations as determined from the HMBC spectrum allowed the connectivity of the structural fragments around each methyl group to be deduced. The configurations of all double bonds were confirmed from a NOESY experiment on **5**. The presence of a key NOESY correlation between the vinylic H-11 (δ_H_ 5.08) and H-13b (δ_H_ 2.04) made it possible to identify the configuration of the olefin at C-11/C-12 as *E* geometry. The geometries of the conjugated diene at C-1/C-2 and C-3/C-4 were assigned as *E* and *E* on the basis of the crucial NOESY correlations between H-2 (δ_H_ 6.07)/Me-16 (δ_H_ 1.06), H-2/Me-18 (δ_H_ 1.76), and H-3 (δ_H_ 5.98)/H-5a (δ_H_ 2.28). The absence of NOESY correlation between H-2 and H-3 and the large coupling constant (*J*_2,3_ = 10.8 Hz) further suggested the *s-trans* geometry of the conjugated double bonds [4]. Based on the similarity of the crucial NOESY correlations, the absolute configurations at C-7 and C-8 of **5** were assumed to be identical with those of compounds **1**–**3**. The ^1^H and ^13^C NMR spectroscopic data (Table 1 and Table 2) of **5** were in full agreement with those of an 7*R*,8*S*-epoxy enantiomer, which had been prepared as an intermediate in an enantioselective total synthesis of (–)-7,8-epoxy-7,8-dihydrocembrene-C [14]. However, the specific rotation [α]^25^_D_ +40.0 (*c* 0.5, CHCl_3_) of **5** was substantially different from that of the 7*R*,8*S* enantiomer, [α]^20^_D_ −41.7 (*c* 0.35, CHCl_3_), suggesting that the absolute configurations of 7,8-epoxy group in **5** should be 7*S* and 8*R*. The full assignments of ehrenbergol E (**5**) have been disclosed (Table 1 and Table 2) by extensive 2D NMR spectroscopic data analyses for the first time.

Macrocyclic cembranoids and their derivatives, distributed mainly in the soft corals belonging to the genus *Sarcophyton* (Alcyoniidae), have attracted much attention from chemists due to their structural complexity and remarkable biological activities such as antitumor [15,16,17,18,19,20,21], ichthyotoxic [22], anti-inflammatory [23], anti-osteoporotic [24], neuroprotective [25], antiangiogenic [26], antimetastatic [26], and antibacterial properties [27]. Our previous studies have reported that macrocyclic cembranoids possess antiviral and cytotoxic properties [4,5,6], which prompted us to evaluate the cytotoxicity against P-388, A-459, HT-29, and HEL cells as well as antiviral activity against HCMV cells of compounds **1**–**5**.

Preliminary cytotoxic screening revealed that **4** and **5** displayed cytotoxicity against P-388 cell line with EC_50_ values of 2.0 and 3.0 μM, respectively. However, the other tested compounds were not cytotoxic to P-388, A-549, HT-29 and HEL cells. Compounds **1**–**5** were examined for antiviral activity against human cytomegalovirus (HCMV) using a human embryonic lung (HEL). At a concentration of 1 μg/mL, compound **2** showed slight antiviral activity against HCMV cells with an IC_50_ value of 45.0 μg/mL. With the exception of the above findings, the obtained negative results showed that the others exhibited no discernible activity against HCMV cells (IC_50_ > 50 μg/mL). According to our studies, the cembrane-type derivatives endowed with various biological activities and *S. ehrenbergi* identified as a potential marine source for the discovery of promising new drugs. Advanced bioactivity assays and chemical modifications for these compounds will be carried out if sufficient material can be recollected from the marine organism.

## 3. Experimental Section

### 3.1. General Experimental Procedures

Optical rotations were determined with a JASCO P1020 digital polarimeter (Tokyo, Japan). Ultraviolet (UV) and infrared (IR) spectra were obtained on a JASCO V-650 (Tokyo, Japan) and JASCO FT/IR-4100 spectrometer (Tokyo, Japan), respectively. CD analysis was performed on a JASCO J-815 spectropolarimeter (Tokyo, Japan). The NMR spectra were recorded on a Varian MR 400 NMR spectrometer (Santa Clara, CA, USA) at 400 MHz for ^1^H and 100 MHz for ^13^C, respectively. Chemical shifts are expressed in δ (ppm) referring to the solvent peaks δ_H_ 7.15 and δ_C_ 128.5 for C_6_D_6_, and δ_H_ 7.27 and δ_C_ 77.0 for CDCl_3_, respectively, and coupling constants are expressed in Hz. ESIMS were recorded by ESI FT-MS on a Bruker APEX II mass spectrometer (Bremen, Germany). Silica gel 60 (Merck, Darmstadt, Germany, 230–400 mesh), Sephadex LH-20 (Amersham Pharmacia Biotech., Piscataway, NJ, USA), and LiChroprep RP-18 (Merck, 40–63 μm) were used for column chromatography. Precoated silica gel plates (Merck, Kieselgel 60 F_254_, 0.25 mm) and precoated RP-18 F_254s_ plates (Merck, 1.05560) were used for TLC analyses. High-performance liquid chromatography (HPLC) was performed on a Hitachi L-7100 pump (Tokyo, Japan) equipped with a Hitachi L-7400 UV detector at 220 nm and a semi-preparative reversed-phase column (Merck, Hibar Purospher RP-18e, 5 μm, 250 × 10 mm).

### 3.2. Animal Material

Specimens of *S. ehrenbergi* were collected at the San-Hsian-Tai, Taitong County, Taiwan, in July 2009, at a depth of 6 m, and were frozen immediately after collection. Identification was kindly verified by Prof. Chang-Feng Dai, Institute of Oceanography, National Taiwan University (Taipei, Taiwan). A voucher specimen (ST-13) is available for inspection at the Department of Marine Biotechnology and Resources, National Sun Yat-sen University, Kaohsiung, Taiwan.

### 3.3. Extraction and Isolation

The frozen material (4.0 kg) was cut into small pieces and extracted exhaustively with acetone (4 × 3 L) at room temperature. The combined acetone extracts were concentrated to a brown gum, which was partitioned between H_2_O and EtOAc. The resulting EtOAc extract was concentrated under reduced pressure to give a dark brown residue (46.9 g), which was fractionated by gradient Si-60 gel column chromatography eluting with a step gradient (0%–100% EtOAc in *n*-hexane) to yield 20 fractions. Fraction 14 (3.6 g) eluted with *n*-hexane–EtOAc (2:1) was subjected to column chromatography on silica gel using *n*-hexane–EtOAc mixtures of increasing polarity for elution to give 10 subfractions. A subfraction 14-4 (0.7 g) eluted with *n*-hexane–EtOAc (6:1) was subjected to a RP-18 gravity column using 60% MeOH in H_2_O, 70% MeOH in H_2_O, 80% MeOH in H_2_O, 90% MeOH in H_2_O, and 100% MeOH, respectively. Altogether, 5 subfractions were obtained, of which subfraction 14-4-3 (80 mg) was further purified by RP-18 HPLC (85% MeOH in H_2_O) to afford **5** (5.1 mg). Similarly, fraction 16 (2.0 g) eluted with *n*-hexane–EtOAc (1:2) was subjected to a silica gel column using *n*-hexane–EtOAc gradient (10:1 to 1:10) for elution to give 11 subfractions. A subfraction 16-3 (108 mg) eluted with *n*-hexane–EtOAc (10:1) was applied to a RP-18 gravity column (MeOH/H_2_O, 50:50 to 100% MeOH) to separate 6 subfractions. Subsequently, a subfraction 16-3-3 (13 mg) was purified by RP-18 HPLC (70% MeOH in H_2_O) to obtain **3** (4.9 mg). A subfraction 16-6 (597 mg) eluted with *n*-hexane–EtOAc (4:1) was chromatographed on a RP-18 gravity column (MeOH/H_2_O, 50:50 to 100% MeOH) to separate 6 subfractions. Subsequently, a subfraction 16-6-2 (13 mg) was purified by RP-18 HPLC (65% MeOH in H_2_O) to give **2** (1.2 mg). A subfraction 16-7 (598 mg) was applied to column chromatography on RP-18 gravity column using 70% MeOH in H_2_O to afford a mixture (109 mg) that was further purified by RP-18 HPLC (70% MeOH in H_2_O) to afford **4** (2.9 mg). Similarly, fraction 17 (1.8 g) eluted with *n*-hexane–EtOAc (1:4) was fractioned by column chromatography on silica gel column using *n*-hexane with increasing amounts of EtOAc, to fractionate 10 subfractions. Then, a subfraction 17-7 (327 mg) eluted with *n*-hexane–EtOAc (1:1) was applied to a RP-18 gravity column (MeOH/H_2_O, 50:50 to 100% MeOH) to give 6 subfractions. A subfraction 17-7-4 (31 mg) was subjected to RP-18 HPLC (80% MeOH in H_2_O) to afford a mixture (3.2 mg) that was further purified by a short silica gel column using *n*-hexane–EtOAc (6:1) to provide **1** (3.3 mg).

(+)-1,15-Epoxy-2-methoxy-12-methoxycarbonyl-11*E*-sarcophytoxide (**1**): Colorless, viscous oil; [α]^25^_D_ +71 (*c* 0.3, CHCl_3_); UV (MeOH) λ_max_ (log ε) 225 (3.83) nm; IR (KBr) *v*_max_ 2962, 1712, 1457, 1373, 1263, 1088, 1027 cm^−1^; ^1^H NMR and ^13^C NMR data, see Table 1 and Table 2; ESIMS *m*/*z* 415 [M + Na]^+^; HRESIMS *m*/*z* 415.2093 [M + Na]^+^ (calcd for C_22_H_32_O_6_Na, 415.2096) (Figures S1–S6).

(+)-2-*epi*-12-Methoxycarbonyl-11*E*-sarcophine (**2**): Colorless, viscous oil; [α]^25^_D_ +56 (*c* 0.1, CHCl_3_); UV (MeOH) λ_max_ (log ε) 224 (3.89) nm; IR (KBr) *v*_max_ 2926, 1751, 1712, 1701, 1636, 1576, 1436, 1386, 1244, 1109, 1125, 1099 cm^−1^; CD (2.67 × 10^−^^4^ M, MeOH) λ_max_ (Δε) 279 (+6.8), 245 (−1.1) and 215 (−2.9) nm; ^1^H NMR and ^13^C NMR data, Table 1 and Table 2; ESIMS *m*/*z* 383 [M + Na]^+^; HRESIMS *m*/*z* 383.1832 [M + Na]^+^ (calcd for C_21_H_28_O_5_Na, 383.1834) (Figures S7–S12).

3,4-Epoxyehrenberoxide A (**3**): Colorless, viscous oil; [α]^25^_D_ +23 (*c* 0.5, CHCl_3_); UV (MeOH) λ_max_ (log ε) 226 (3.85) nm; IR (KBr) *v*_max_ 2951, 1715, 1643, 1461, 1376, 1179, 1279, 1240, 1195, 1073 cm^−1^; ^1^H NMR and ^13^C NMR data, see Table 1 and Table 2; ESIMS *m*/*z* 401 [M + Na]^+^; HRESIMS *m*/*z* 401.2301 [M + Na]^+^ (calcd for C_22_H_34_O_5_Na, 401.2304) (Figures S13–S18).

Ehrenbergol D (**4**): Colorless, viscous oil; [α]^25^_D_ +35 (*c* 0.3, CHCl_3_); UV (MeOH) λ_max_ (log ε) 245 (3.67) nm; IR (KBr) *v*_max_ 3422, 2959, 1747, 1621, 1438, 1383, 1219, 1041 cm^−1^; CD (9.76 × 10^−^^4^ M, MeOH) λ_max_ (Δε) 259 (+24.9) and 205 (–9.0) nm; ^1^H NMR and ^13^C NMR data, see Table 1 and Table 2; ESIMS *m*/*z* 339 [M + Na]^+^; HRESIMS *m*/*z* 339.1938 [M + Na]^+^ (calcd for C_20_H_28_O_3_Na, 339.1936) (Figures S19–S24).

Ehrenbergol E (**5**): Colorless, viscous oil; [α]^25^_D_ +40 (*c* 0.5, CHCl_3_); UV (MeOH) λ_max_ (log ε) 249 (3.76) nm; IR (KBr) *v*_max_ 3441, 2958, 2932, 1619, 1455, 1376, 1240, 1033 cm^−1^; ^1^H NMR and ^13^C NMR data, see Table 1 and Table 2; ESIMS *m*/*z* 327 [M + Na]^+^; HRESIMS *m*/*z* 327.2303 [M + Na]^+^ (calcd for C_20_H_32_O_2_Na, 327.2300) (Figures S25–S30).

### 3.4. Cytotoxicity Assay

Cytotoxicity was determined against P-388 (mouse lymphocytic leukemia), HT-29 (human colon adenocarcinoma), and A-549 (human lung epithelial carcinoma) tumor cells using a modification of the MTT colorimetric method. The provision of the P-388 cell line was supported by John M. Pezzuto, formerly of the Department of Medicinal Chemistry and Pharmacognosy, University of Illinois at Chicago, IL, USA. These HT-29 and A-549 cell lines were purchased from the American Type Culture Collection (Manassas, VA, USA). The anticancer agent mithramycin was used as the positive control and exhibited EC_50_ values of 0.05, 0.06 and 0.07 μM against P-388, A-549 and HT-29, respectively. The experimental details of this assay were carried out according to a previously described procedure [28,29].

### 3.5. Anti-HCMV Assay

To determine the effects of the natural product upon human cytomegalovirus (HCMV) cytopathic effect (CPE), confluent human embryonic lung (HEL) cells grown in 24-well plates were incubated for 1 h in the presence or absence of various concentrations of tested natural product. Ganciclovir was used as a positive control. Then, cells were infected with HCMV at an input of 1000 pfu (plaque forming units) per well of 24-well dish. Antiviral activity is expressed as IC_50_ (50% inhibitory concentration), or compound concentration required to reduce virus induced CPE by 50% after 7 days as compared with the untreated control. To monitor the cell growth upon treating with natural products, an MTT-colorimetric assay was employed [30].

## 4. Conclusions

Five new polyoxygenated cembranoids **1**–**5** were successfully purified from *Sarcophyton ehrenbergi*. Preliminary cytotoxic screening revealed that compounds **1**–**3** were not cytotoxic to P-388, A-549, HT-29 and HEL cells. However, compounds **4** and **5** displayed cytotoxicity against P-388 with EC_50_ values of 2.0 and 3.0 μM, respectively. The result may suggest that the conjugated double bonds at C-1/C-2 and C-3/C-4 are important for the cytotoxicity against P-388. Additionally, at a concentration of 1 μg/mL, compound **2** showed slight antiviral activity against HCMV with an IC_50_ value of 25.0 μg/mL.

## Supplementary Materials

Supplementary materials can be found at: http://www.mdpi.com/1422-0067/16/03/6140/s1.
