# Supplementary material for: Polyoxygenated Cembrane Diterpenoids from the Soft Coral Sarcophyton ehrenbergi
Source: Int J Mol Sci. 2015 Mar 17;16(3):6140–52. doi: 10.3390/ijms16036140 (PMC4394524; doi:10.3390/ijms16036140)
Supplement: Supplementary file 1 [file ijms-16-06140-s001.pdf]

Supplementary Information

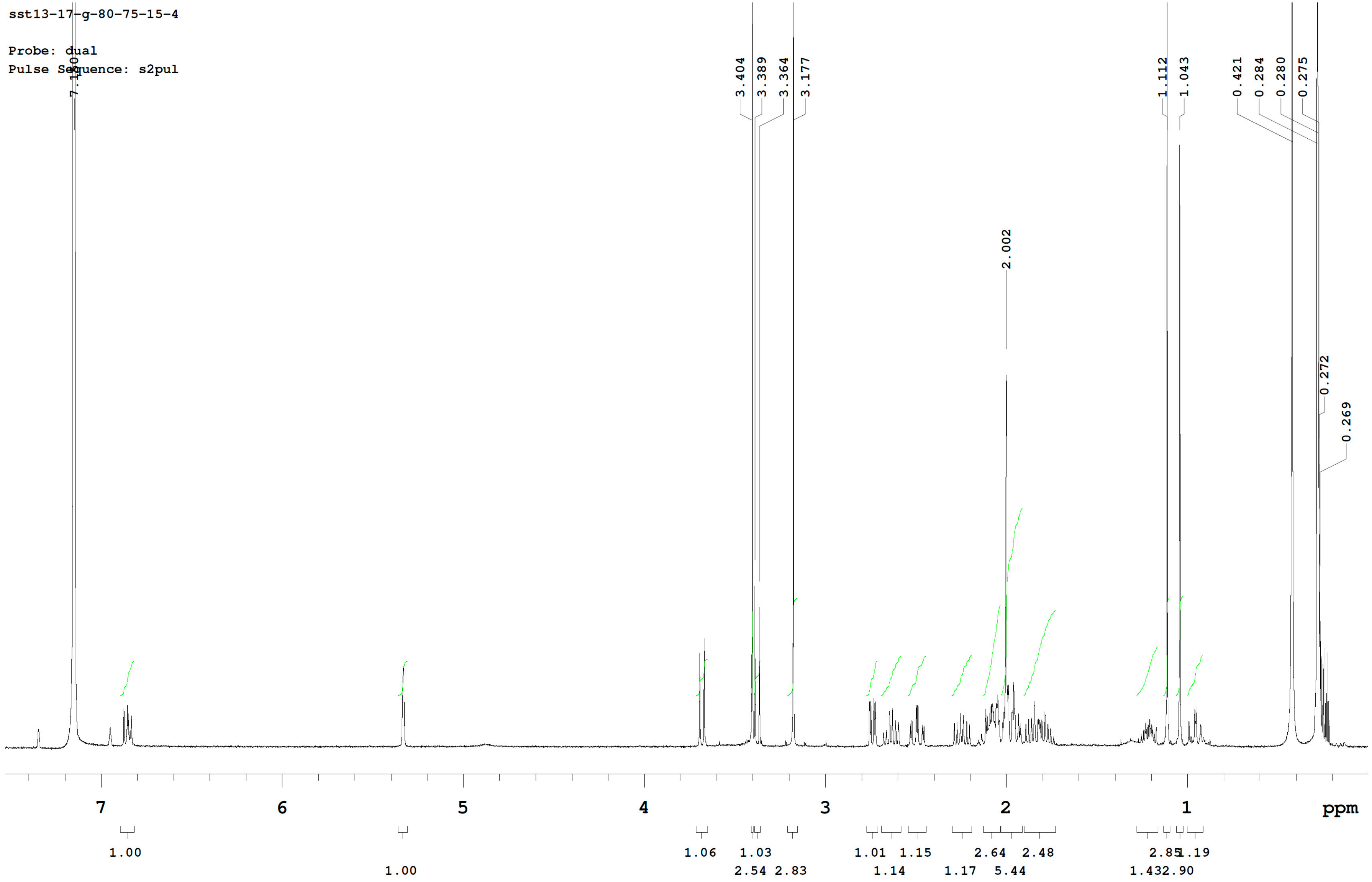

Figure S1. ^{1}H NMR spectrum (400 MHz) of ehrenbergol D (**1**) in C_6D_6.

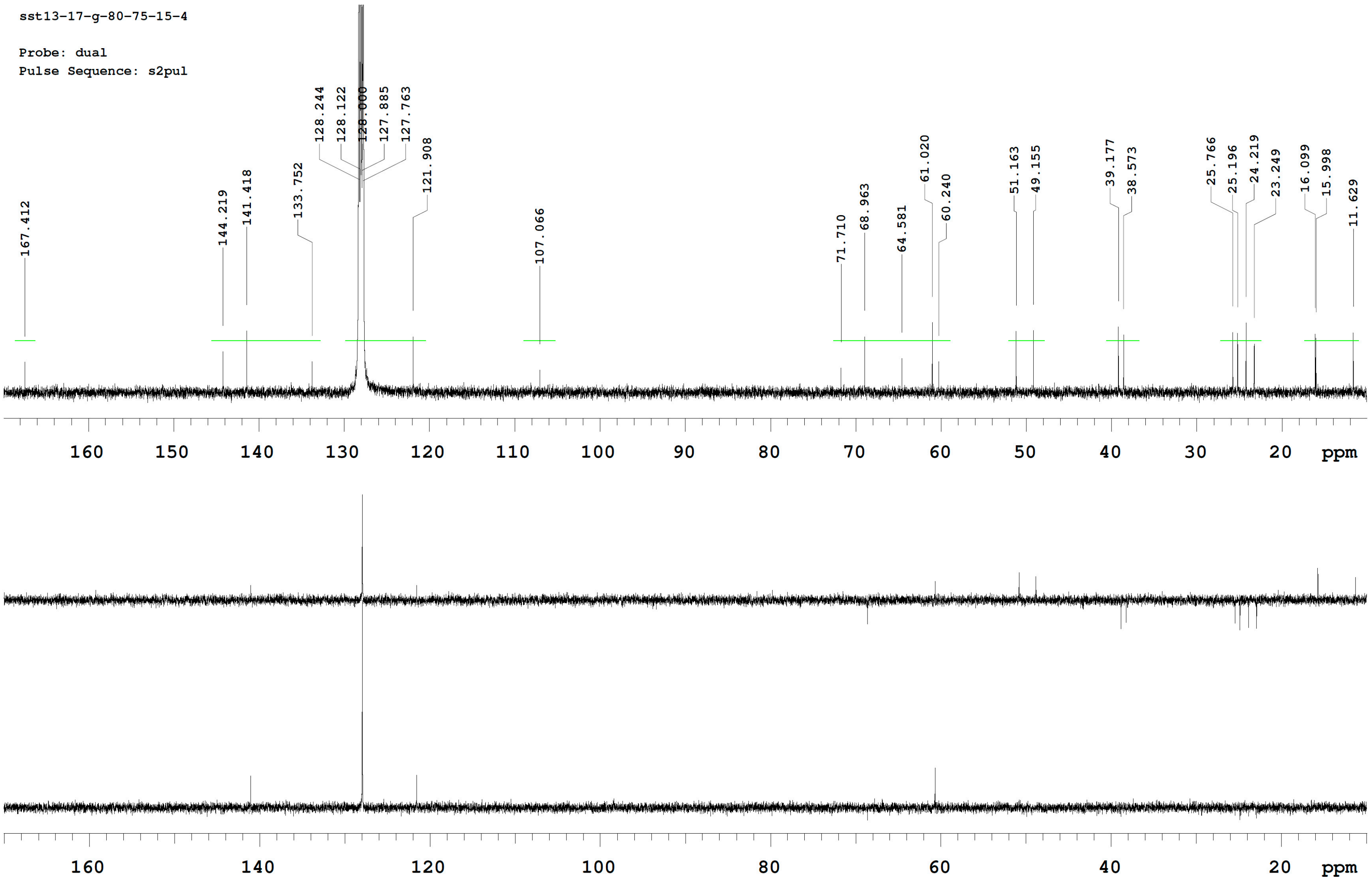

Figure S2. DEPT and ^{13}C NMR spectrum (100 MHz) of ehrenbergol D (**1**) in C_6D_6.

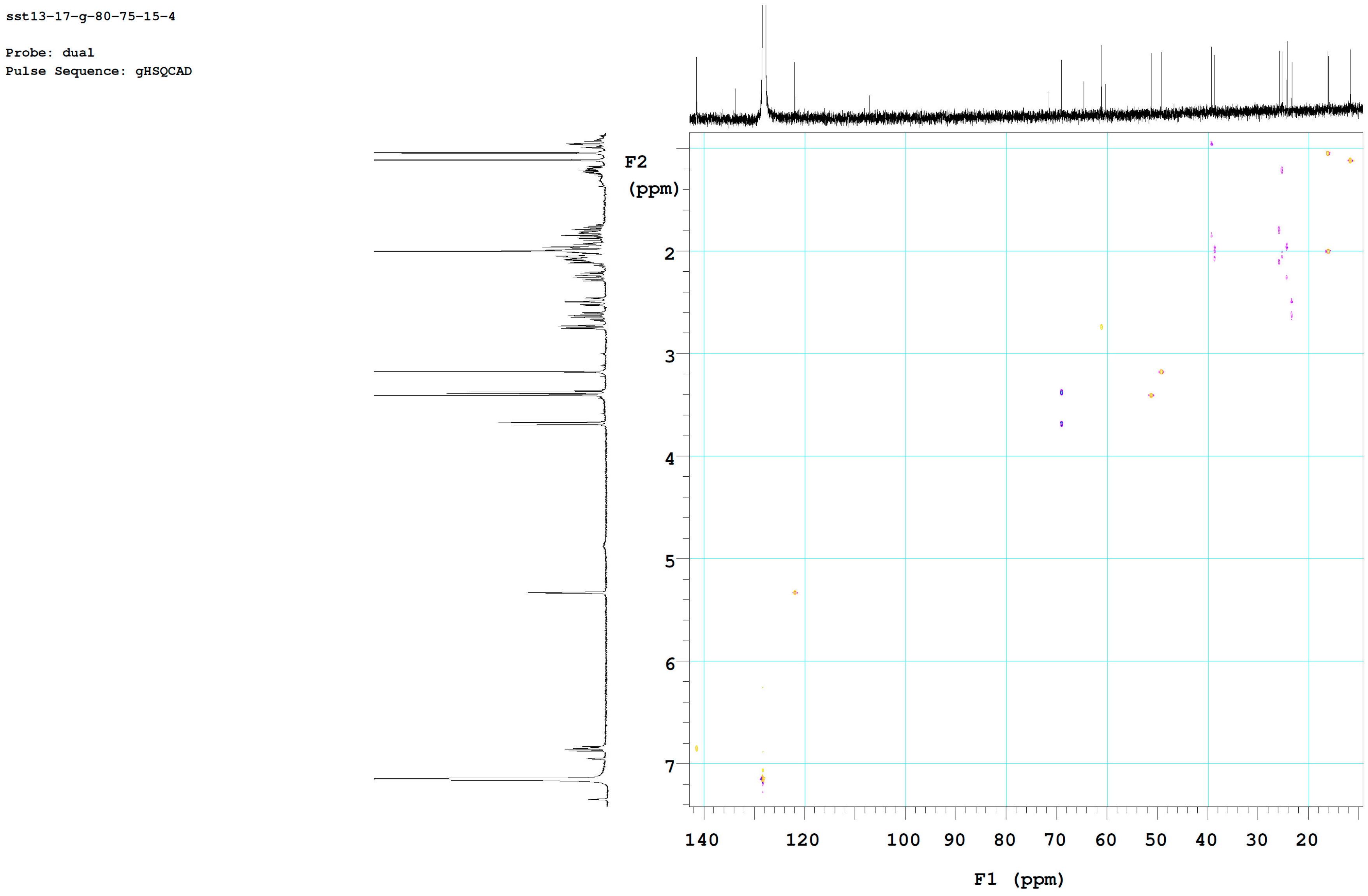

Figure S3. HSQC spectrum (100 MHz) of ehrenbergol D (**1**) in C_6D_6.

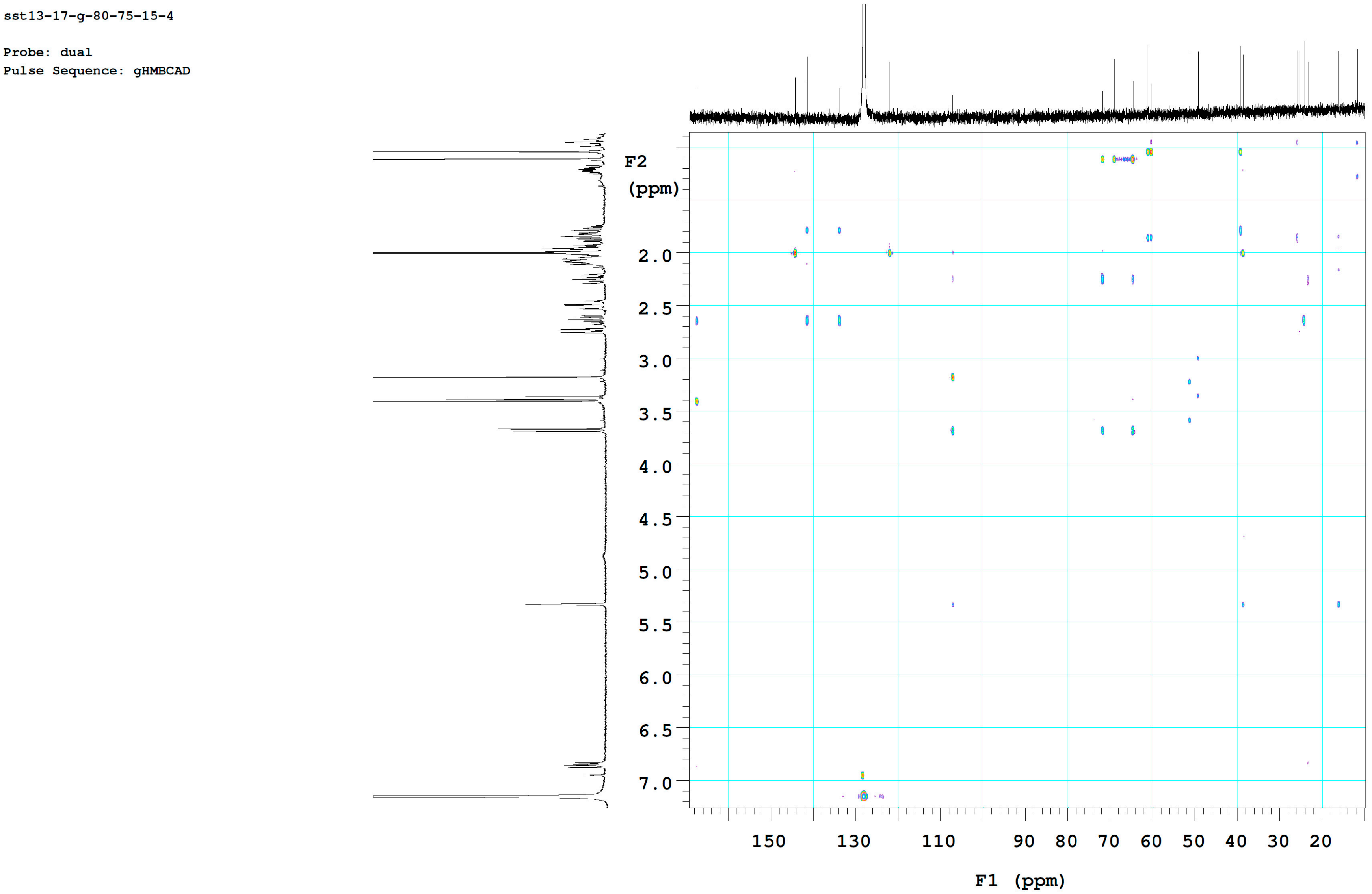

Figure S4. HMBC spectrum (400 MHz) of ehrenbergol D (**1**) in C_6D_6.

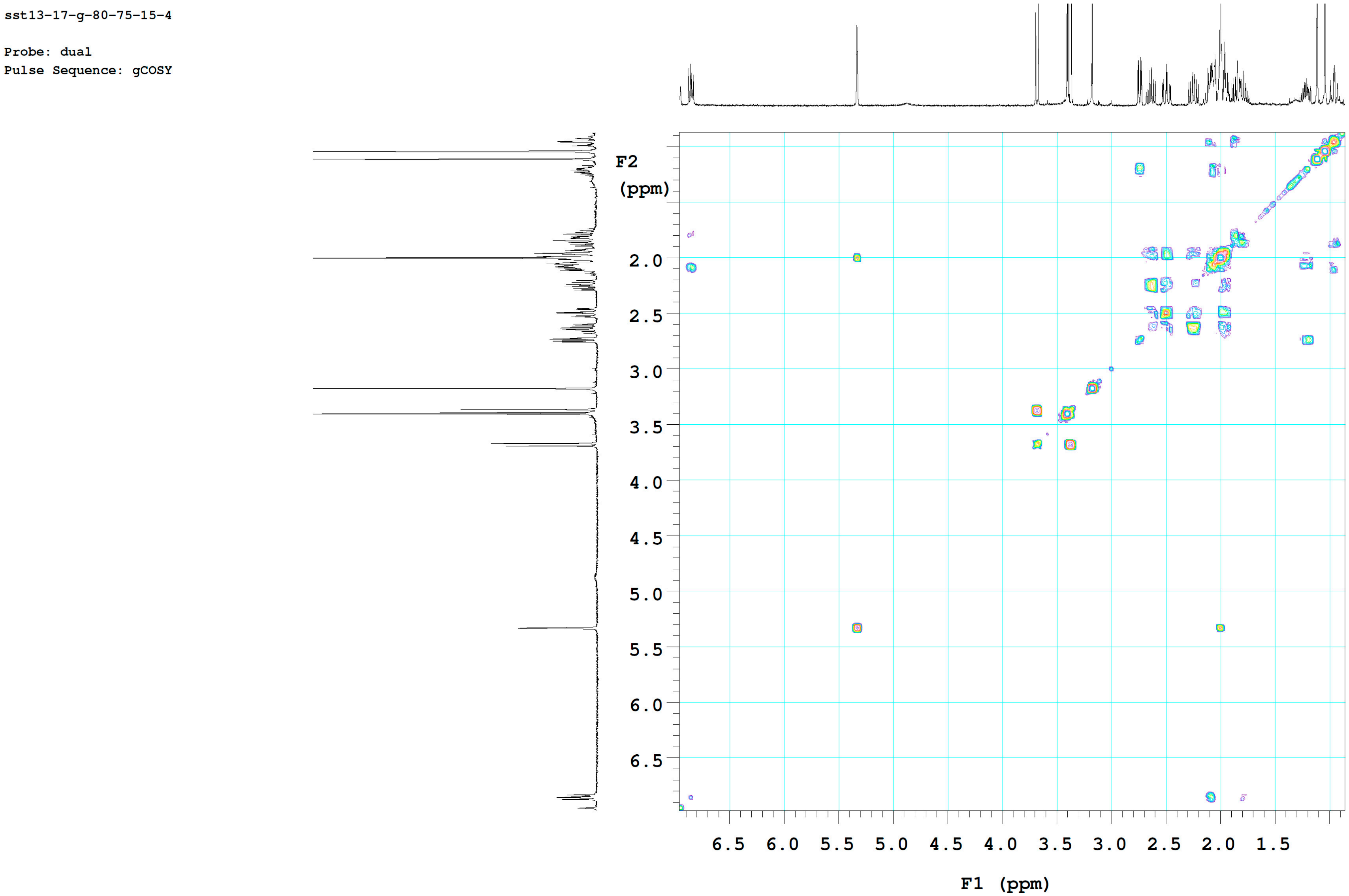

Figure S5. ^1H–^1H COSY spectrum (400 MHz) of ehrenbergol D (**1**) in C_6D_6.

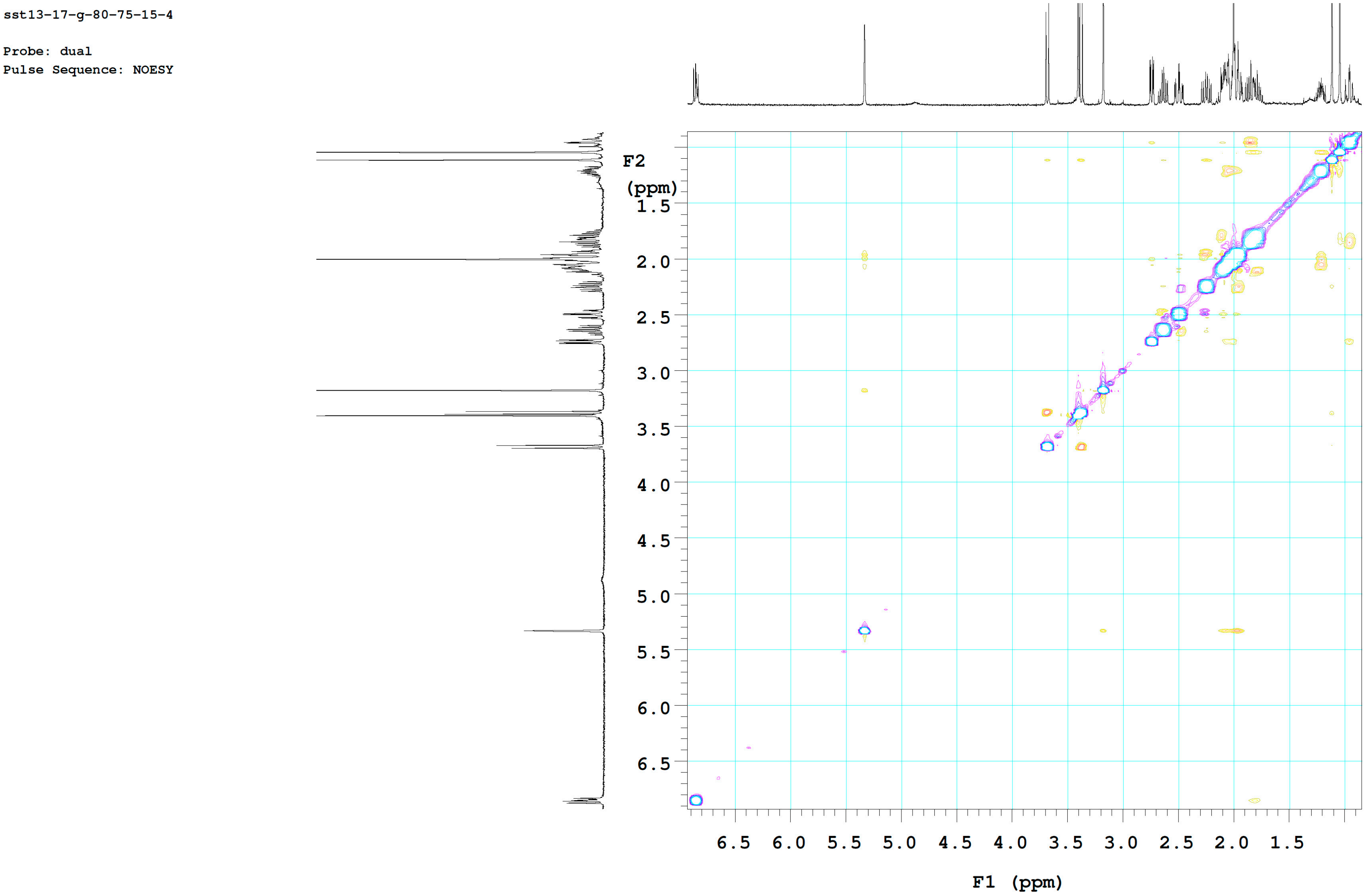

Figure S6. NOESY spectrum (400 MHz) of ehrenbergol D (**1**) in C_6D_6.

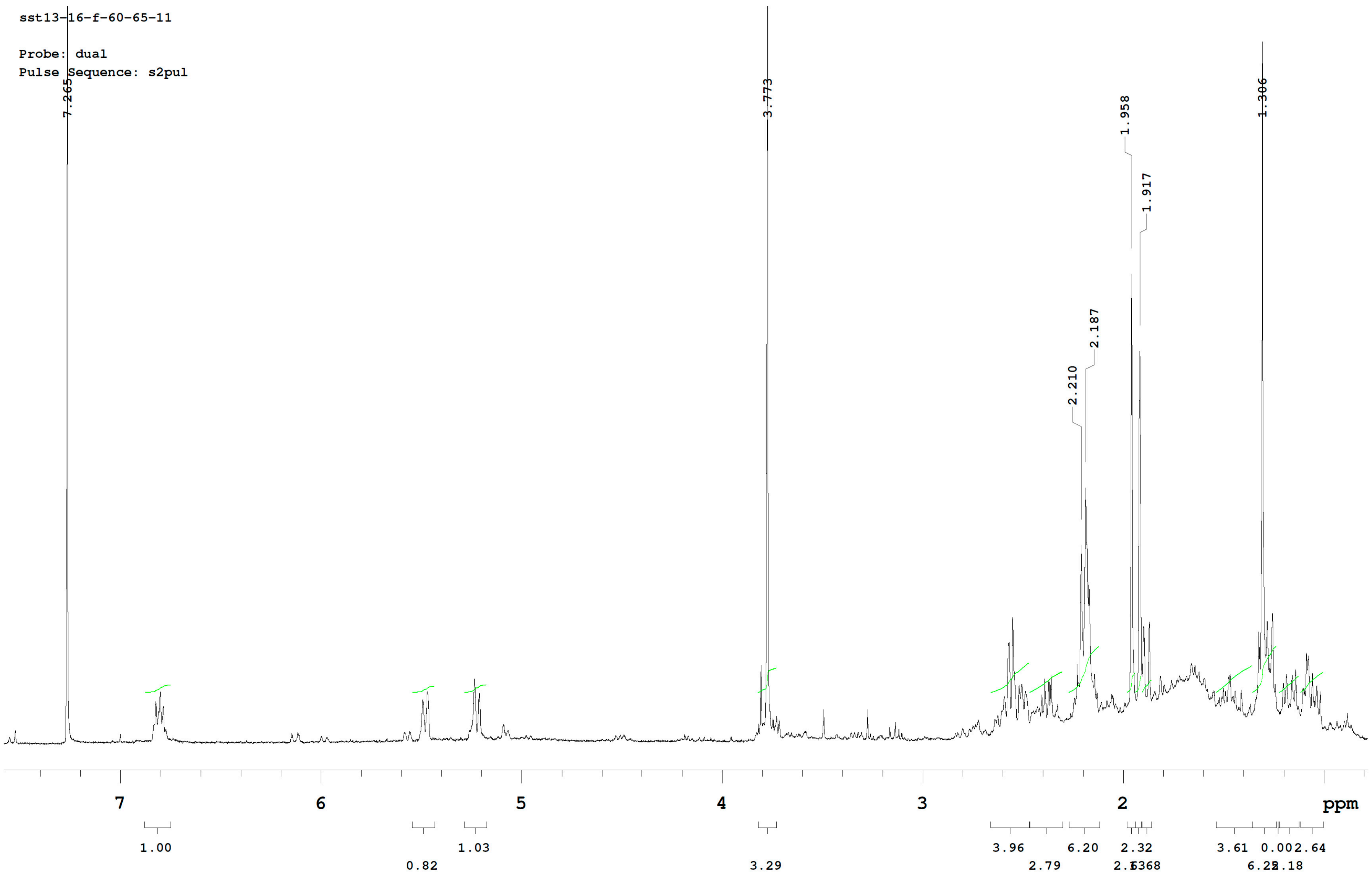

Figure S7. ^{1}H NMR spectrum (400 MHz) of ehrenbergol E (**2**) in $CDCl_3$.

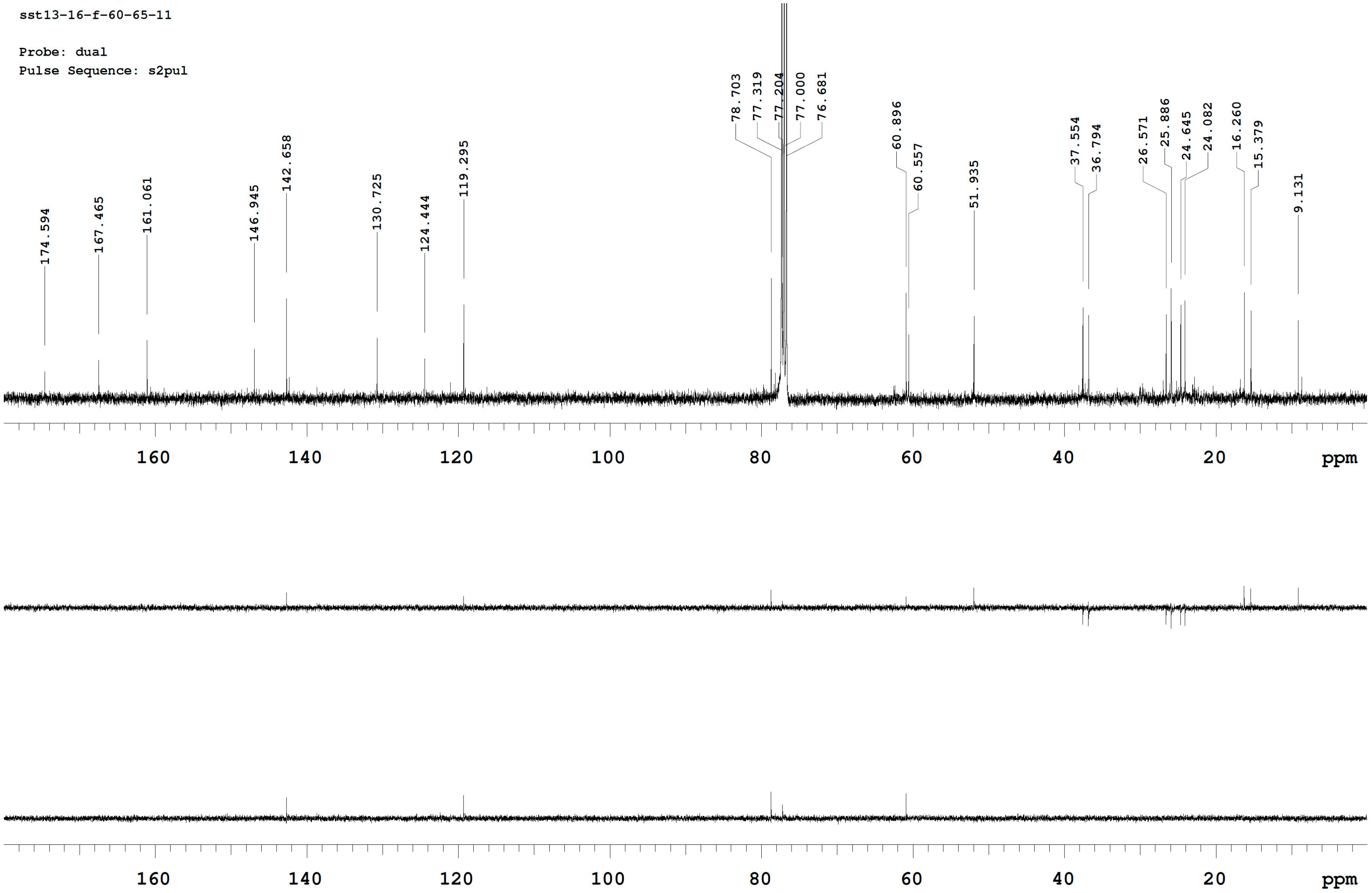

Figure S8. DEPT and ^{13}C NMR spectrum (100 MHz) of ehrenbergol E (**2**) in $CDCl_3$.

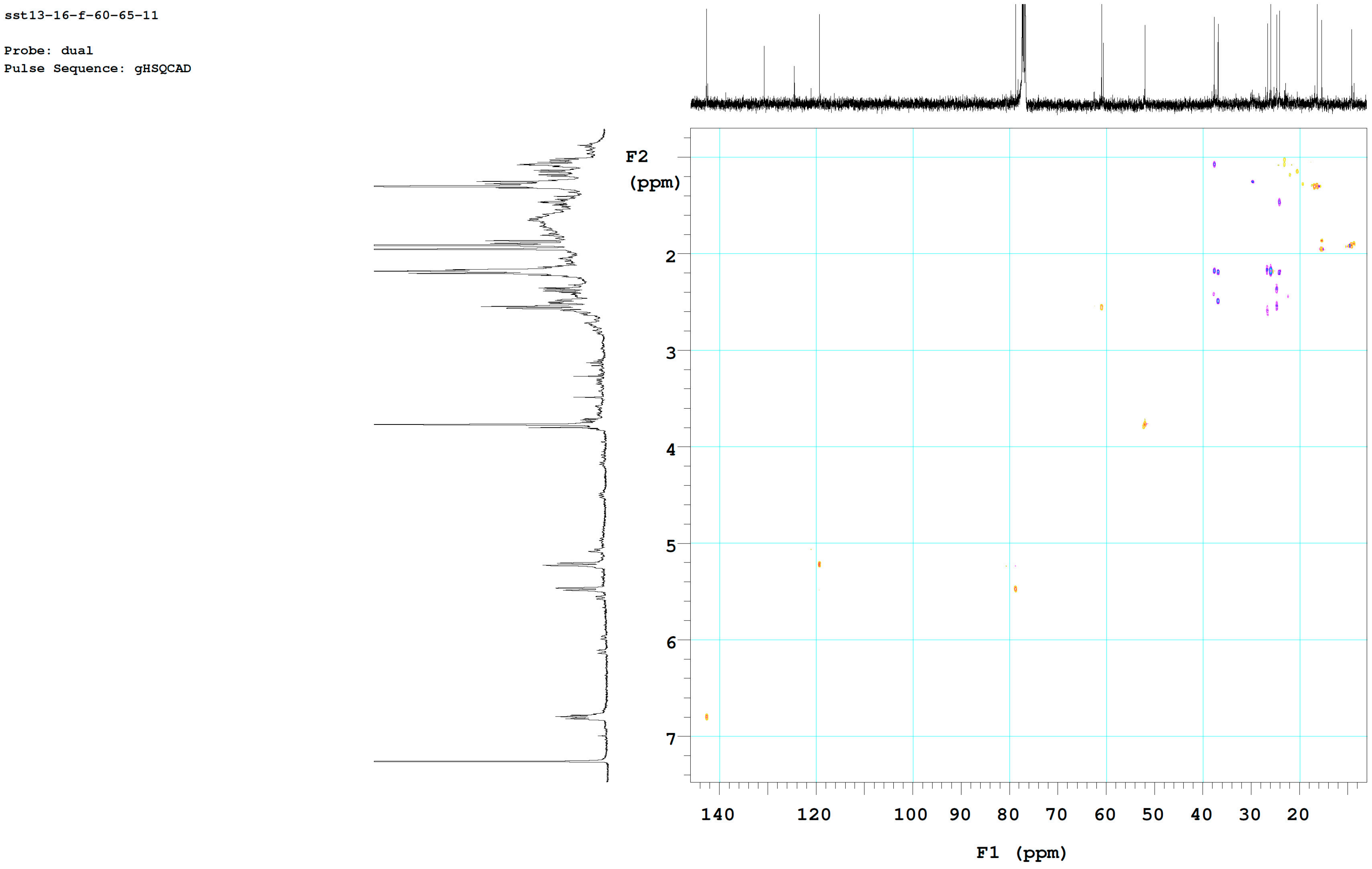

Figure S9. HSQC spectrum (400 MHz) of ehrenbergol E (**2**) in $CDCl_3$.

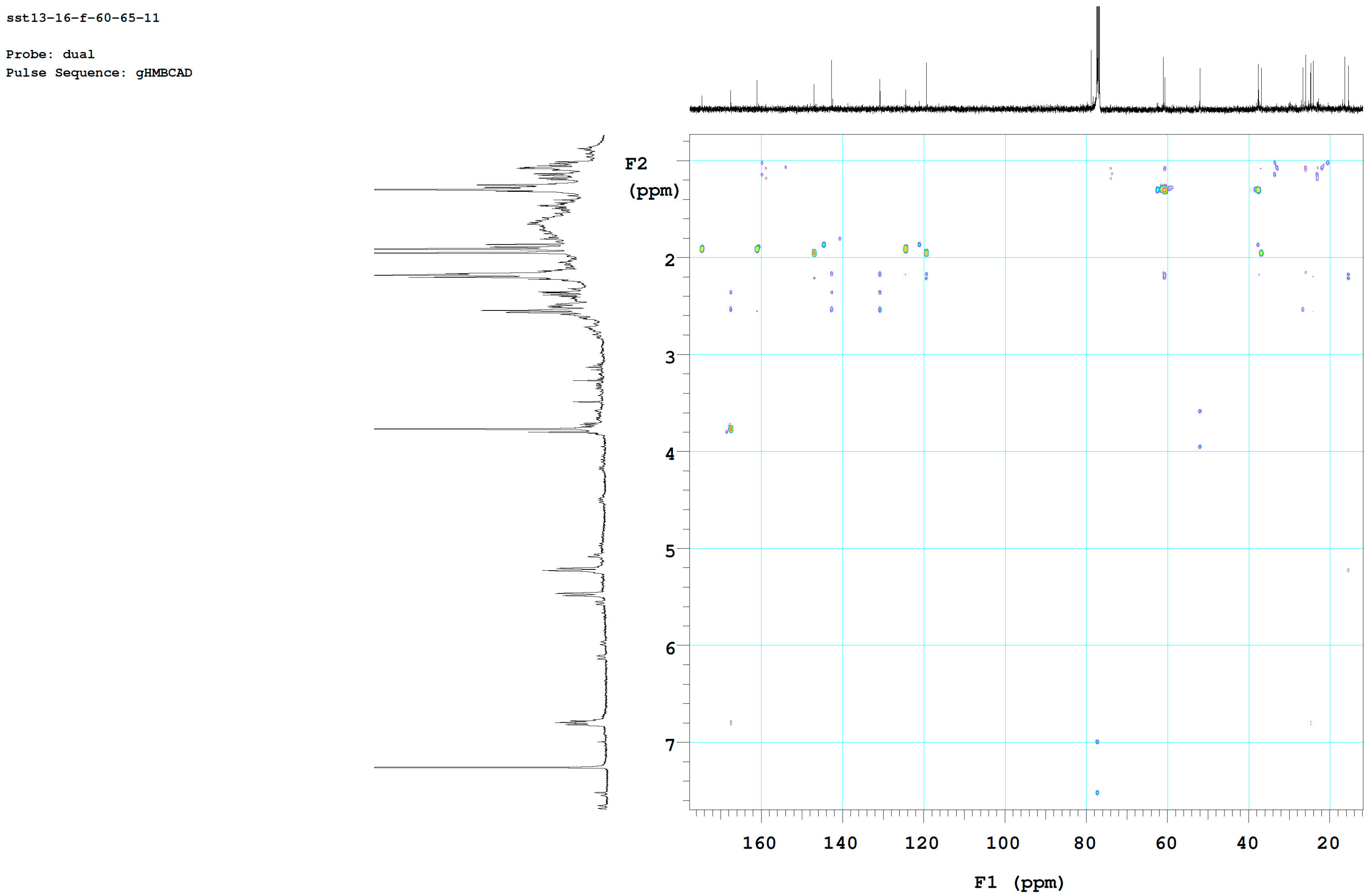

Figure S10. HMBC spectrum (400 MHz) of ehrenbergol E (**2**) in $CDCl_3$.

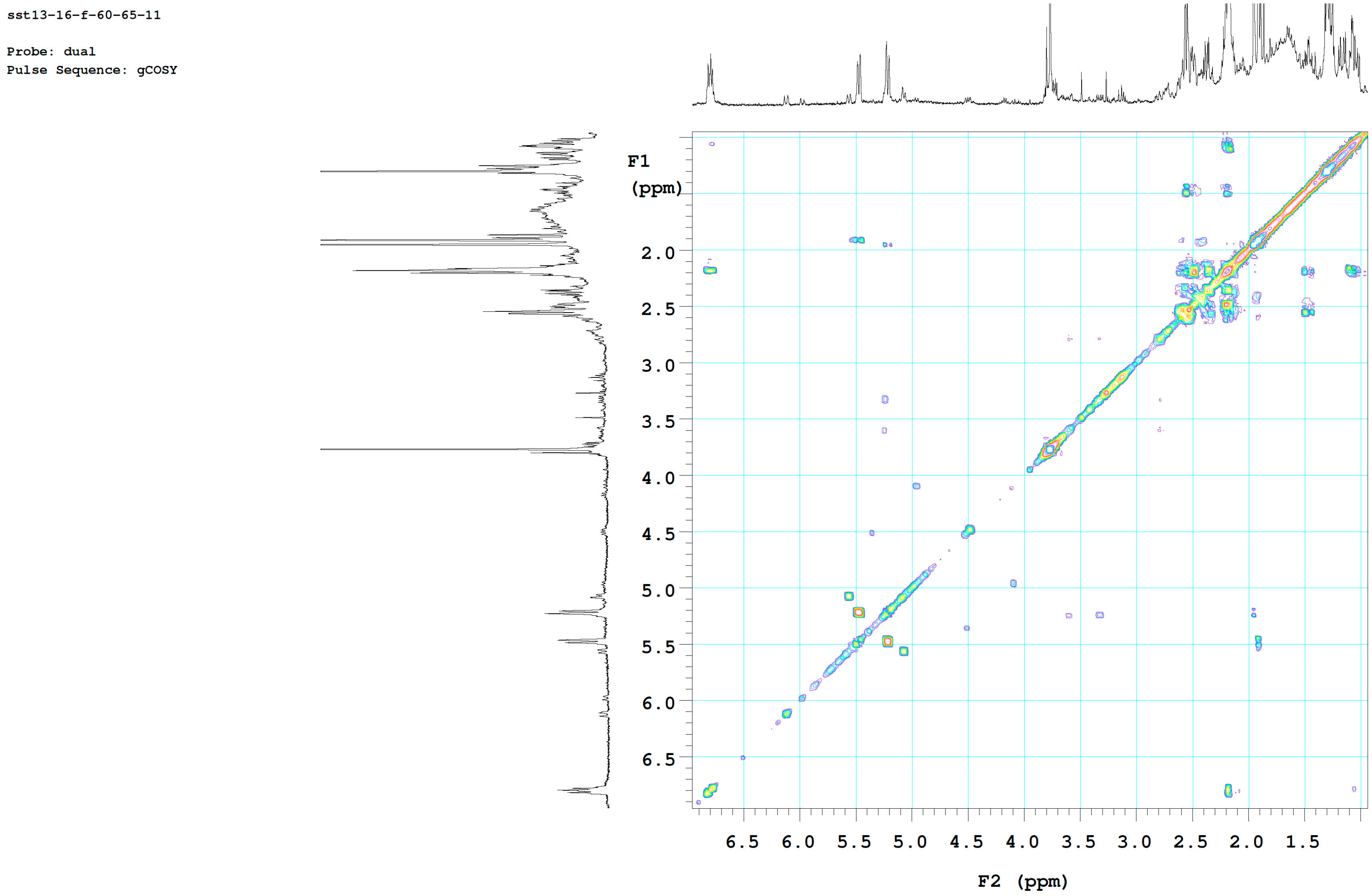

Figure S11. ^{1}H–^{1}H COSY spectrum (400 MHz) of ehrenbergol E (**2**) in $CDCl_3$.

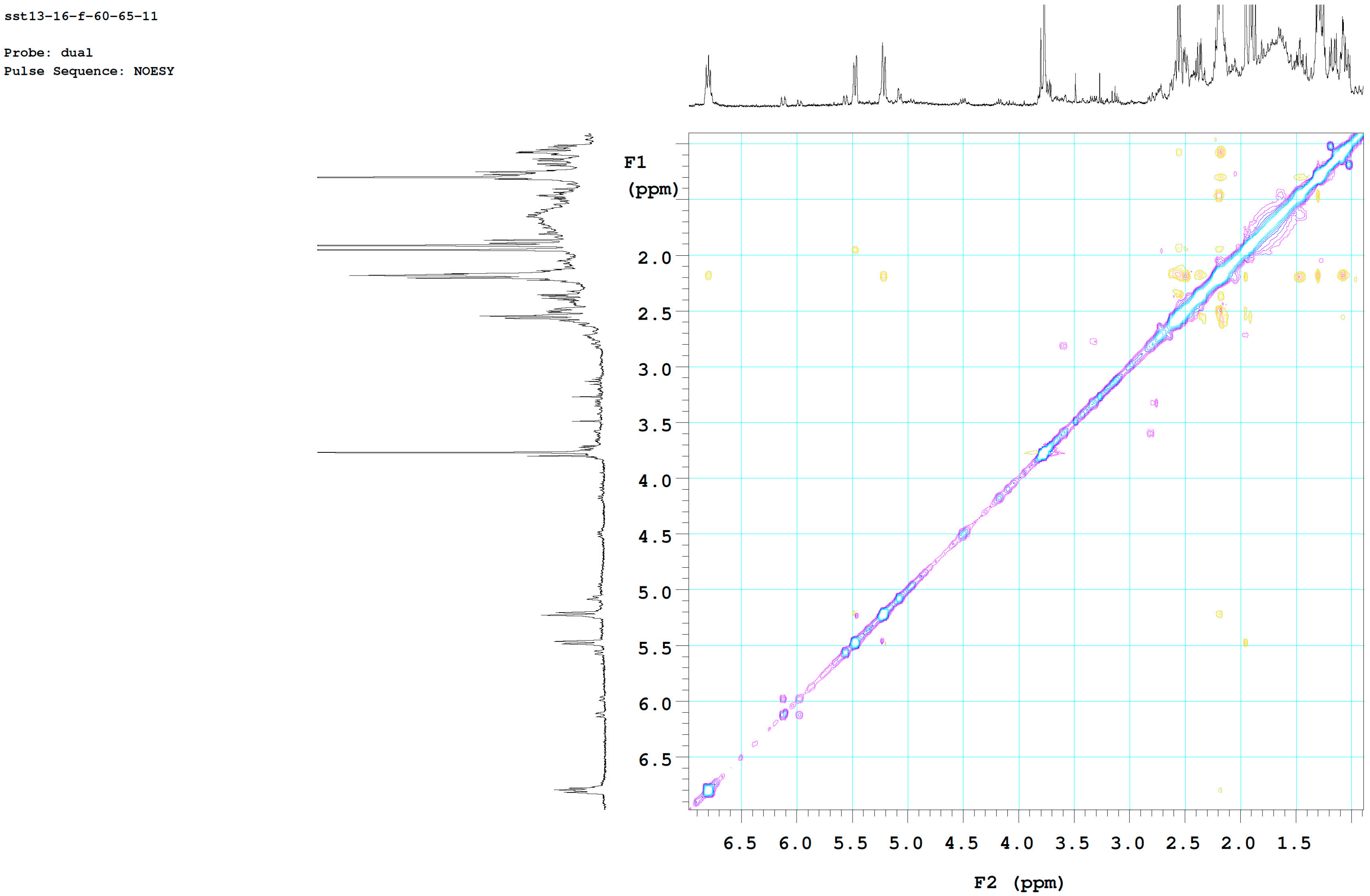

Figure S12. NOESY spectrum (400 MHz) of ehrenbergol E (**2**) in $CDCl_3$.

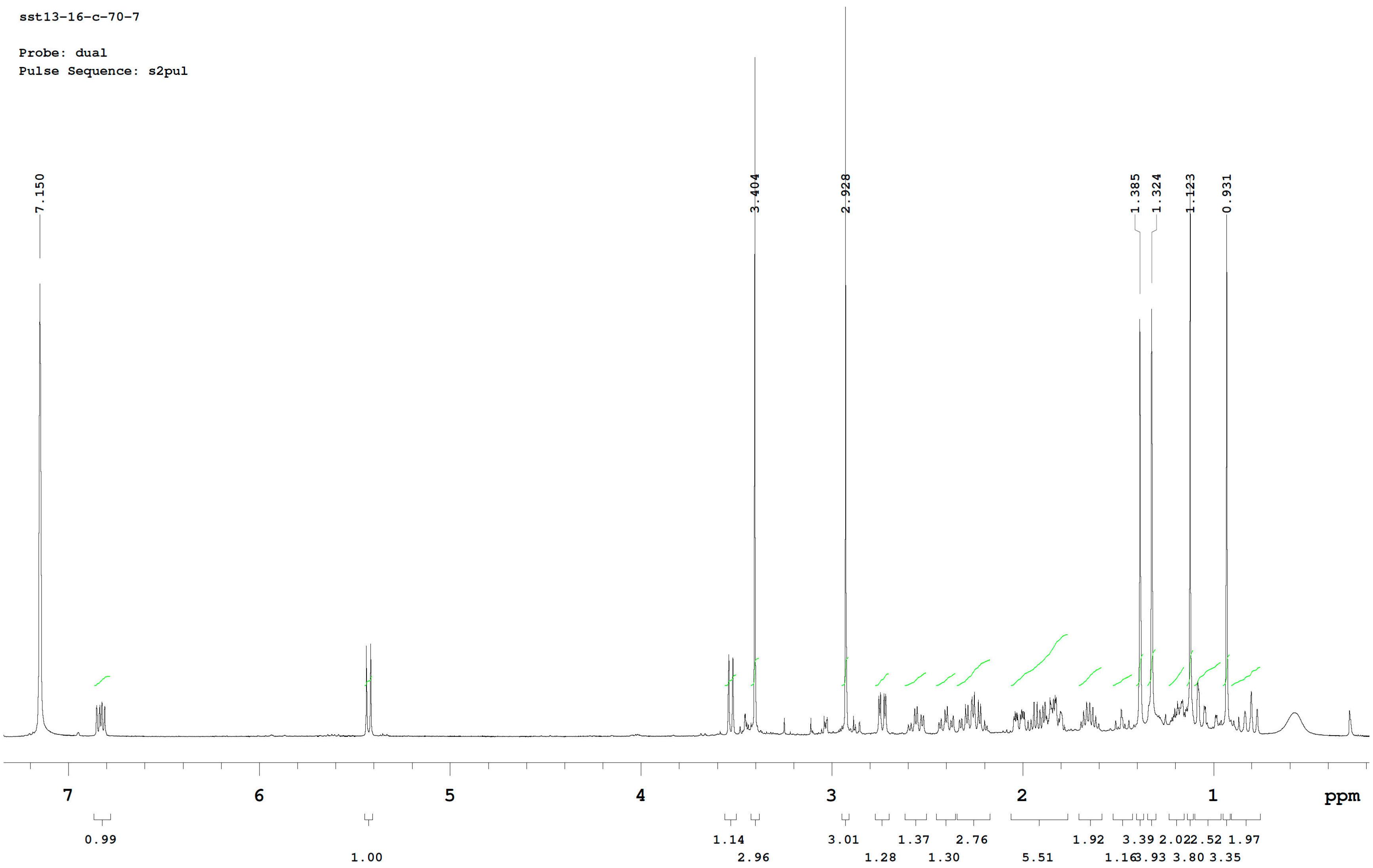

Figure S13. ^{1}H NMR spectrum (400 MHz) of ehrenbergol F (**3**) in C_6D_6.

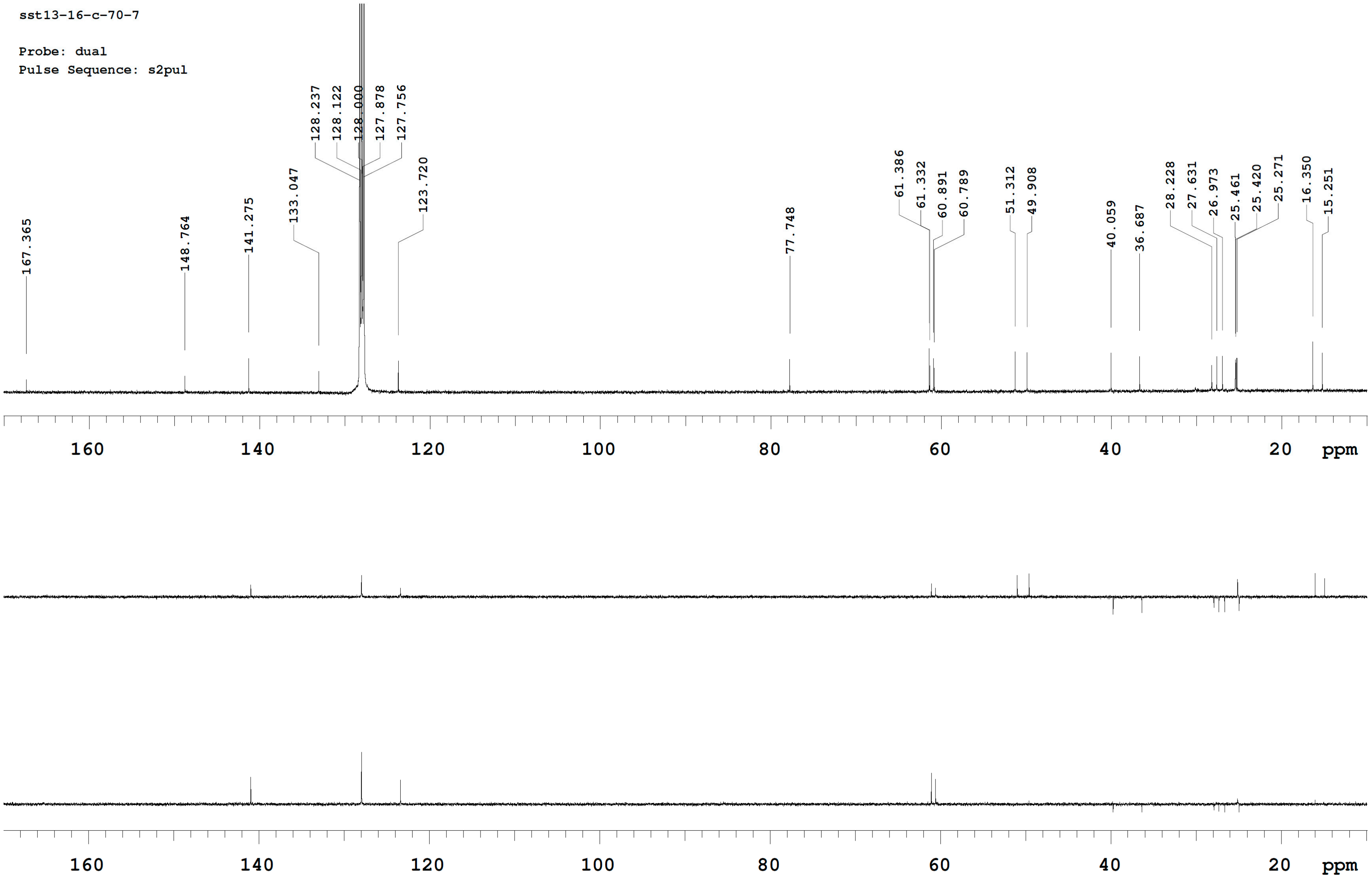

Figure S14. DEPT and ^{13}C NMR spectrum (100 MHz) of ehrenbergol F (**3**) in C_6D_6.

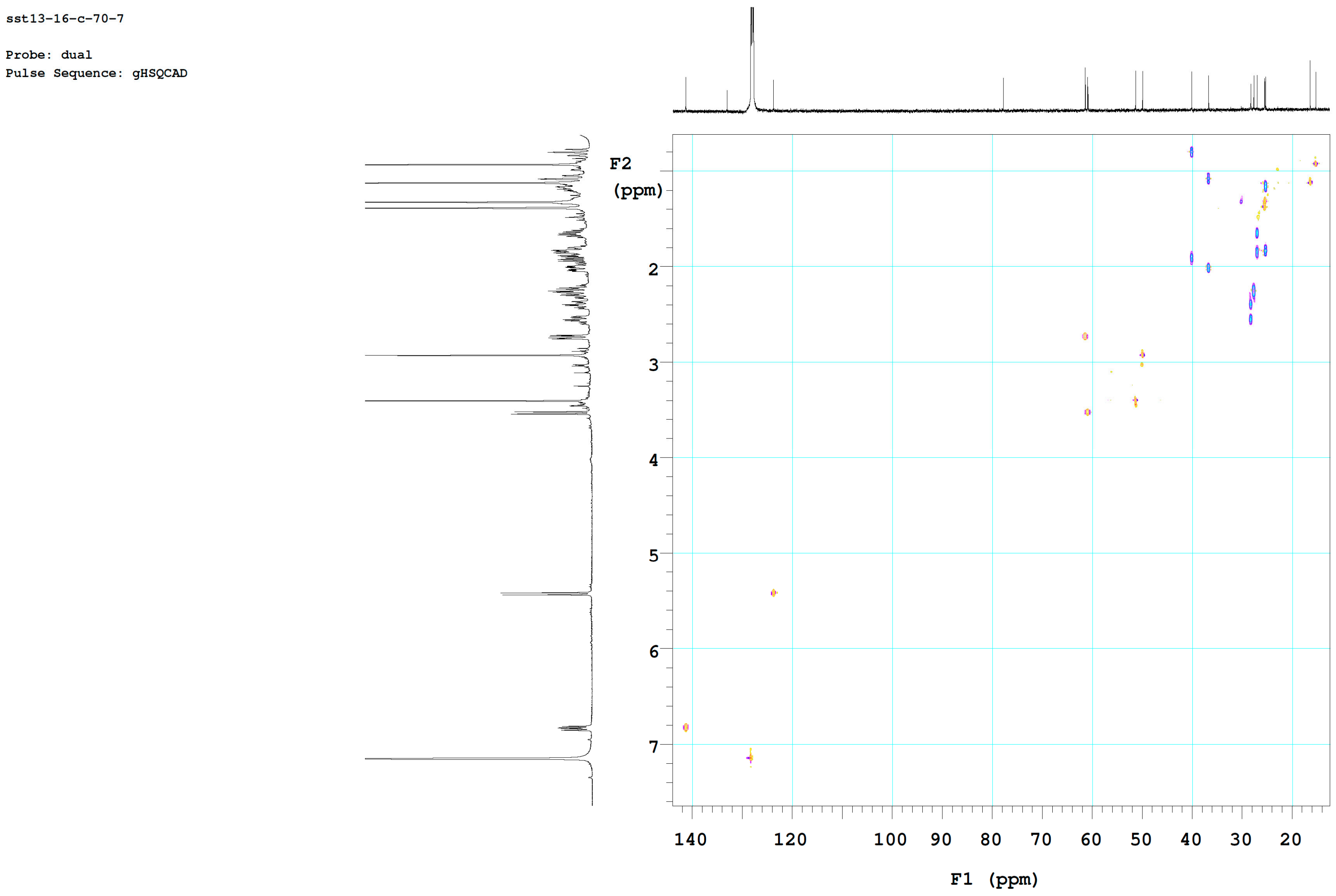

Figure S15. HSQC spectrum (400 MHz) of ehrenbergol F (**3**) in C_6D_6.

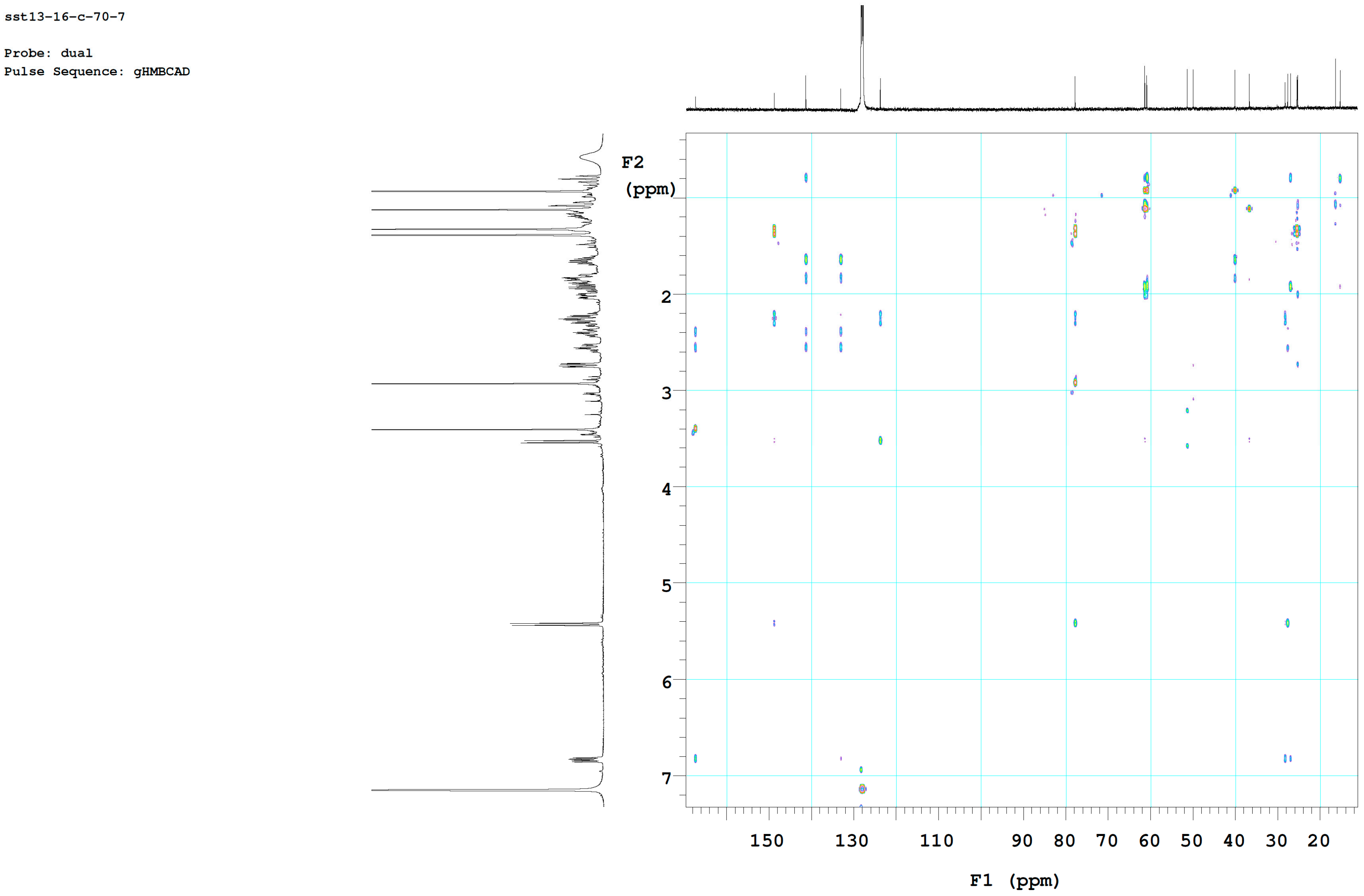

Figure S16. HMBC spectrum (400 MHz) of ehrenbergol F (**3**) in C_6D_6.

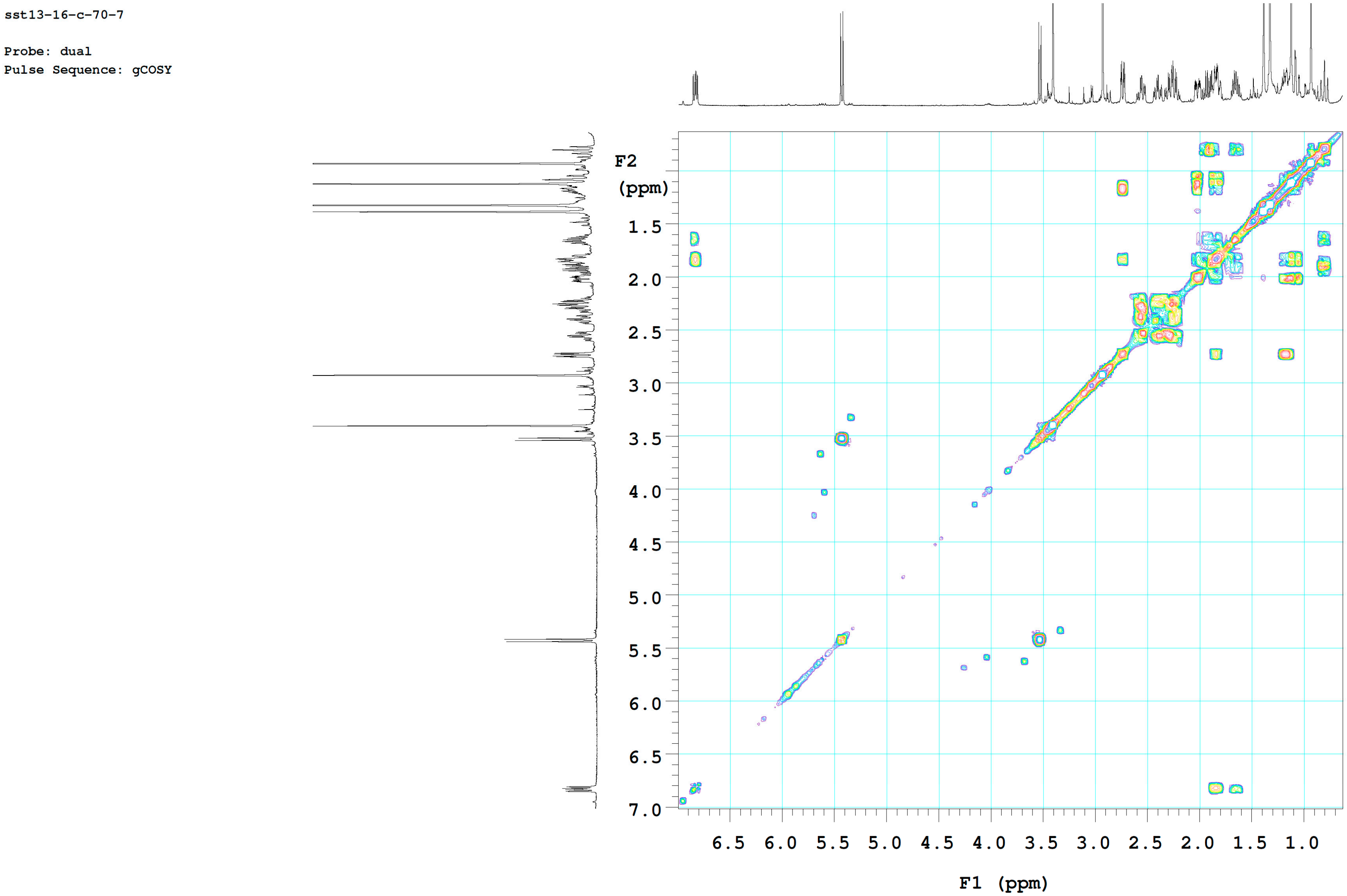

Figure S17. ^{1}H–^{1}H COSY spectrum (400 MHz) of ehrenbergol F (**3**) in C_6D_6.

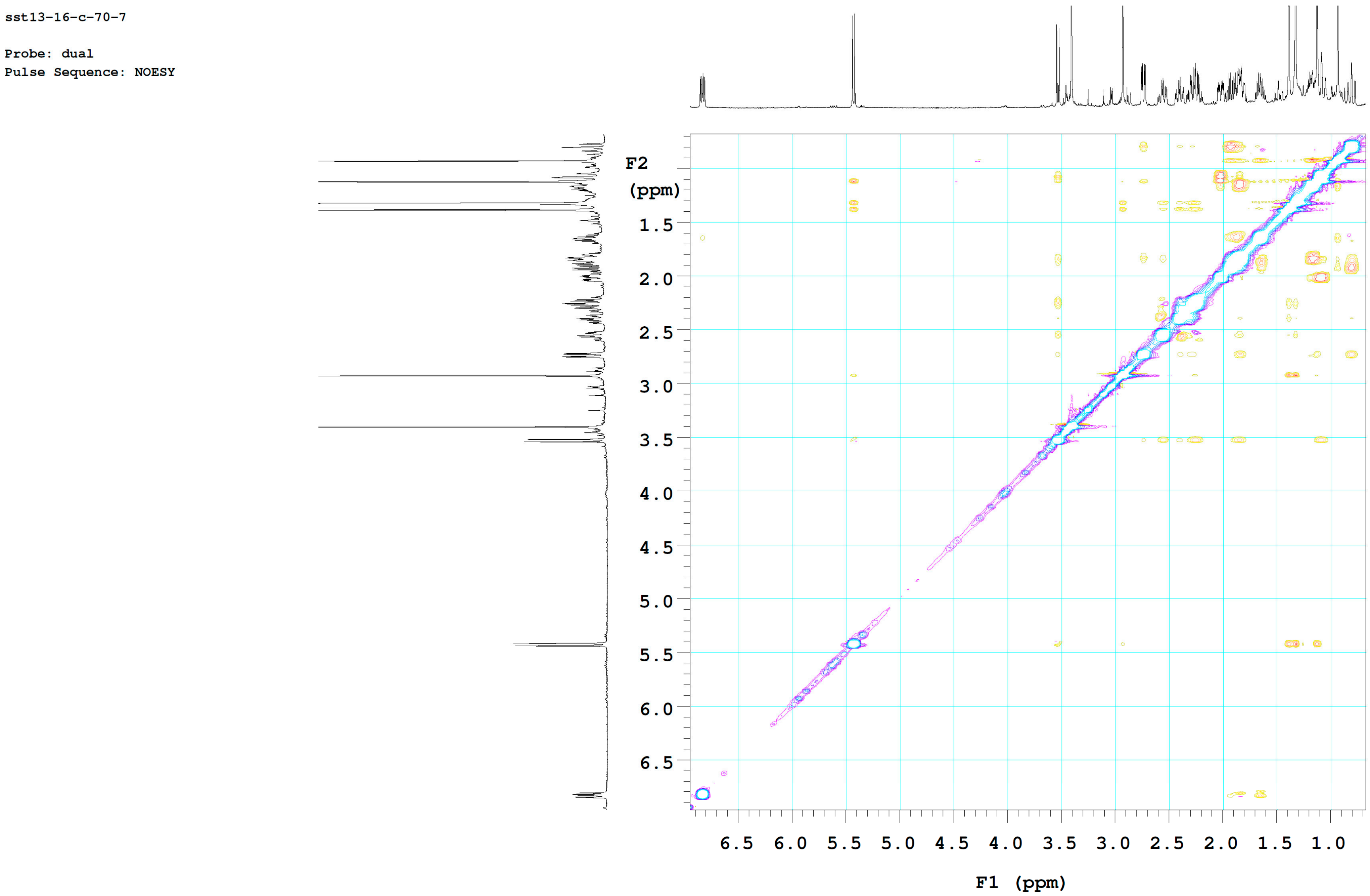

Figure S18. NOESY spectrum (400 MHz) of ehrenbergol F (**3**) in C_6D_6.

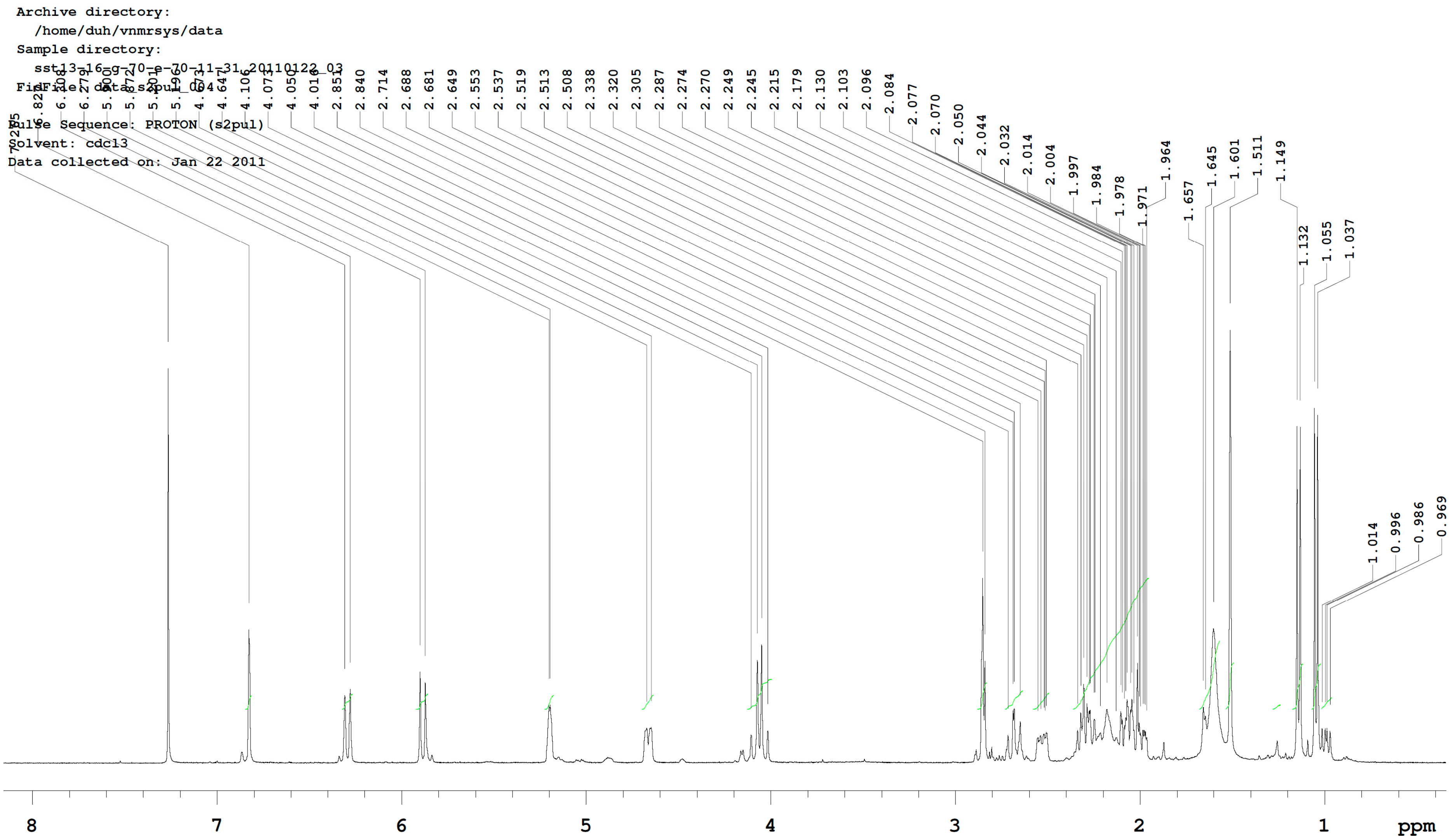

Figure S19. ^{1}H NMR spectrum (400 MHz) of ehrenbergol G (**4**) in C_6D_6.

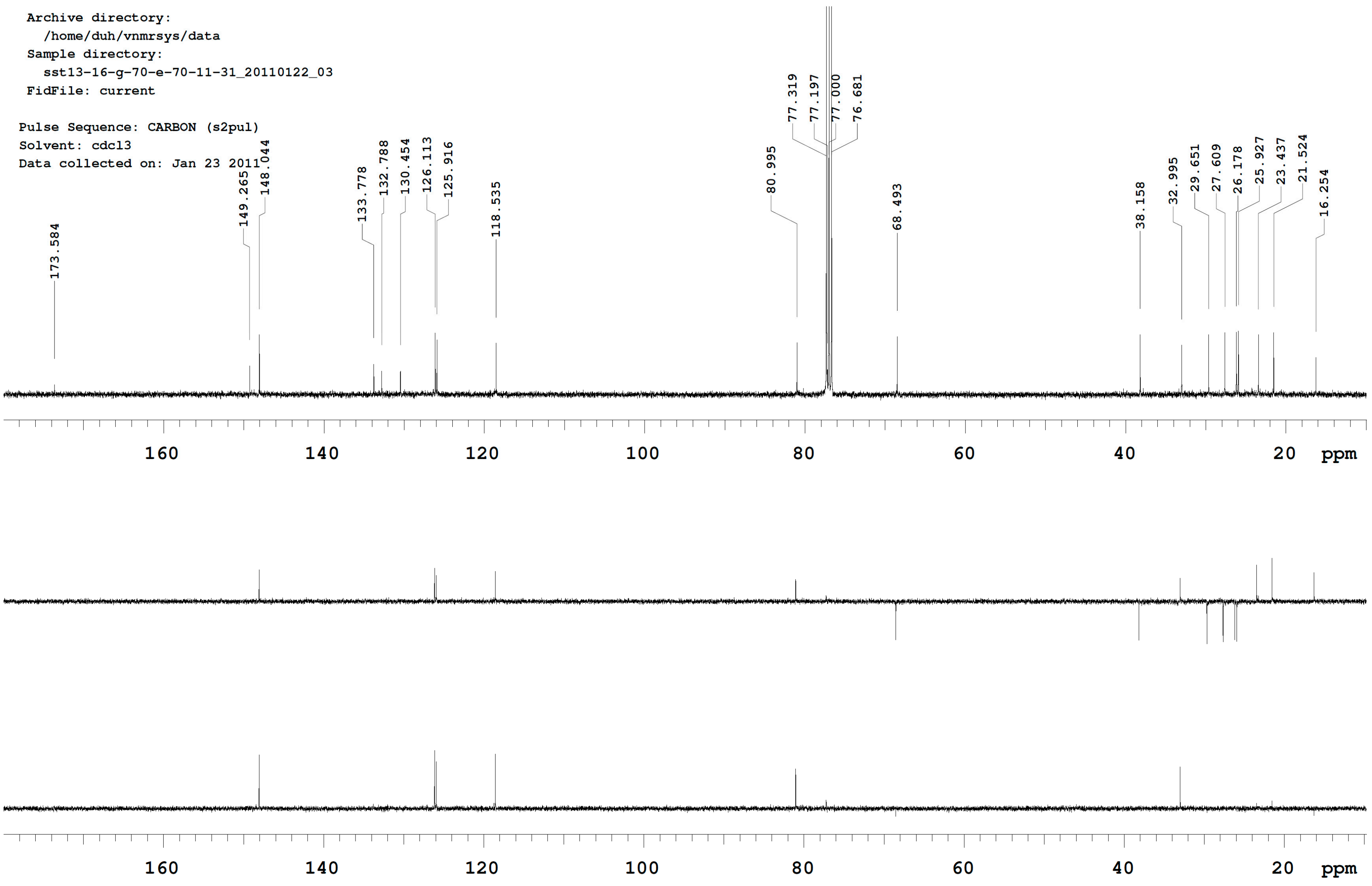

Figure S20. DEPT and ^{13}C NMR spectrum (100 MHz) of ehrenbergol G (**4**) in C_6D_6.

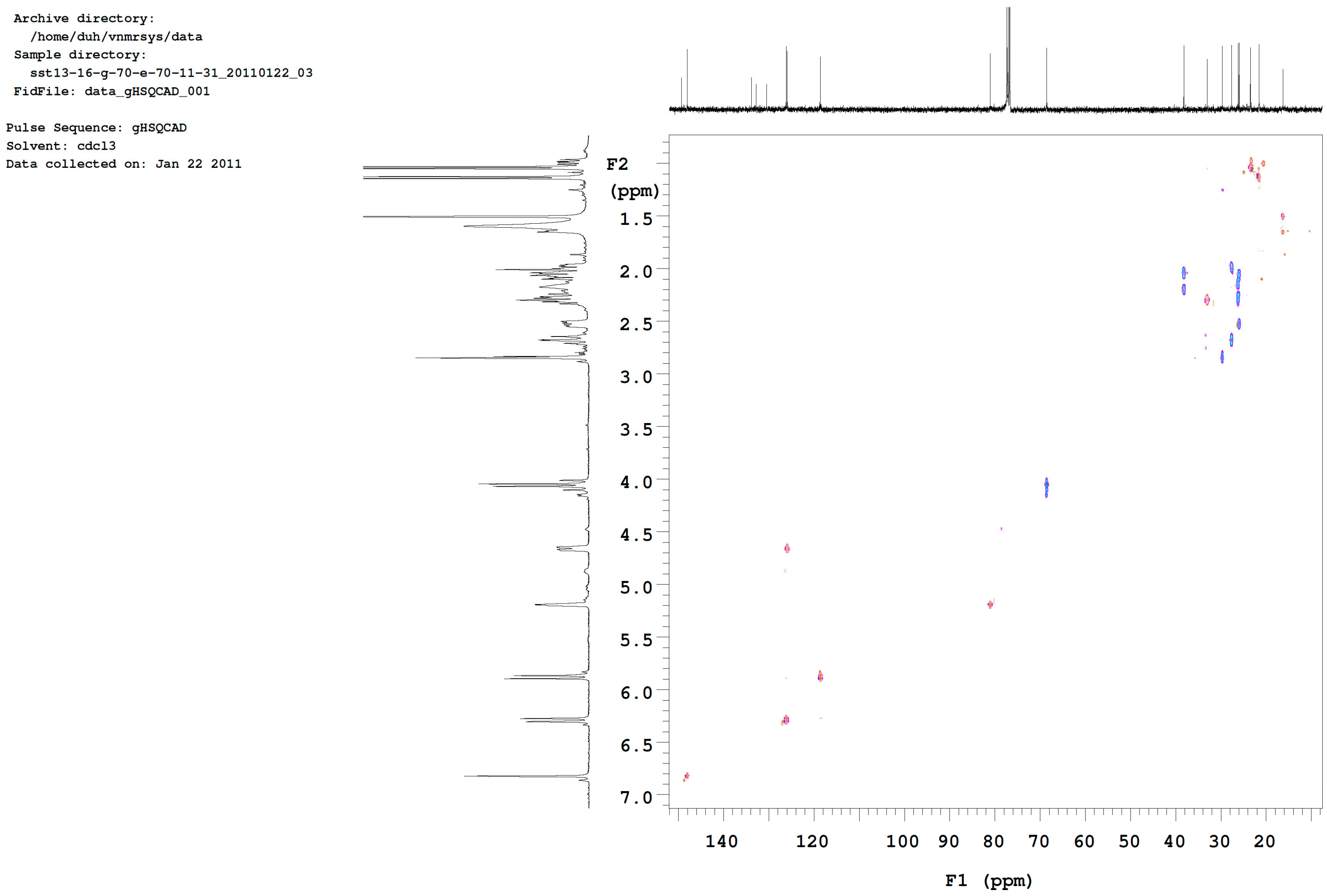

Figure S21. HSQC spectrum (400 MHz) of ehrenbergol G (**4**) in C_6D_6.

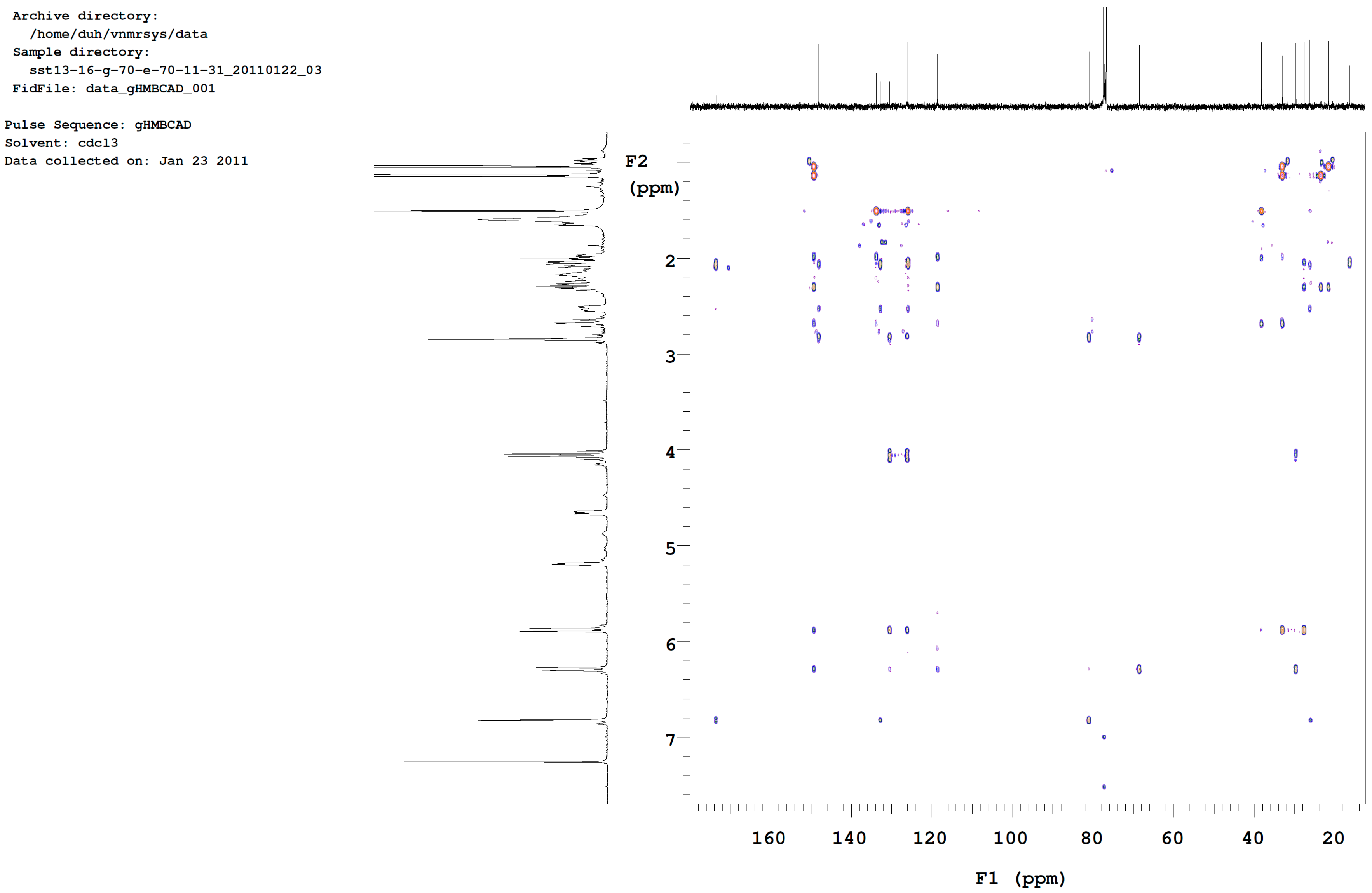

Figure S22. HMBC spectrum (400 MHz) of ehrenbergol G (**4**) in C_6D_6.

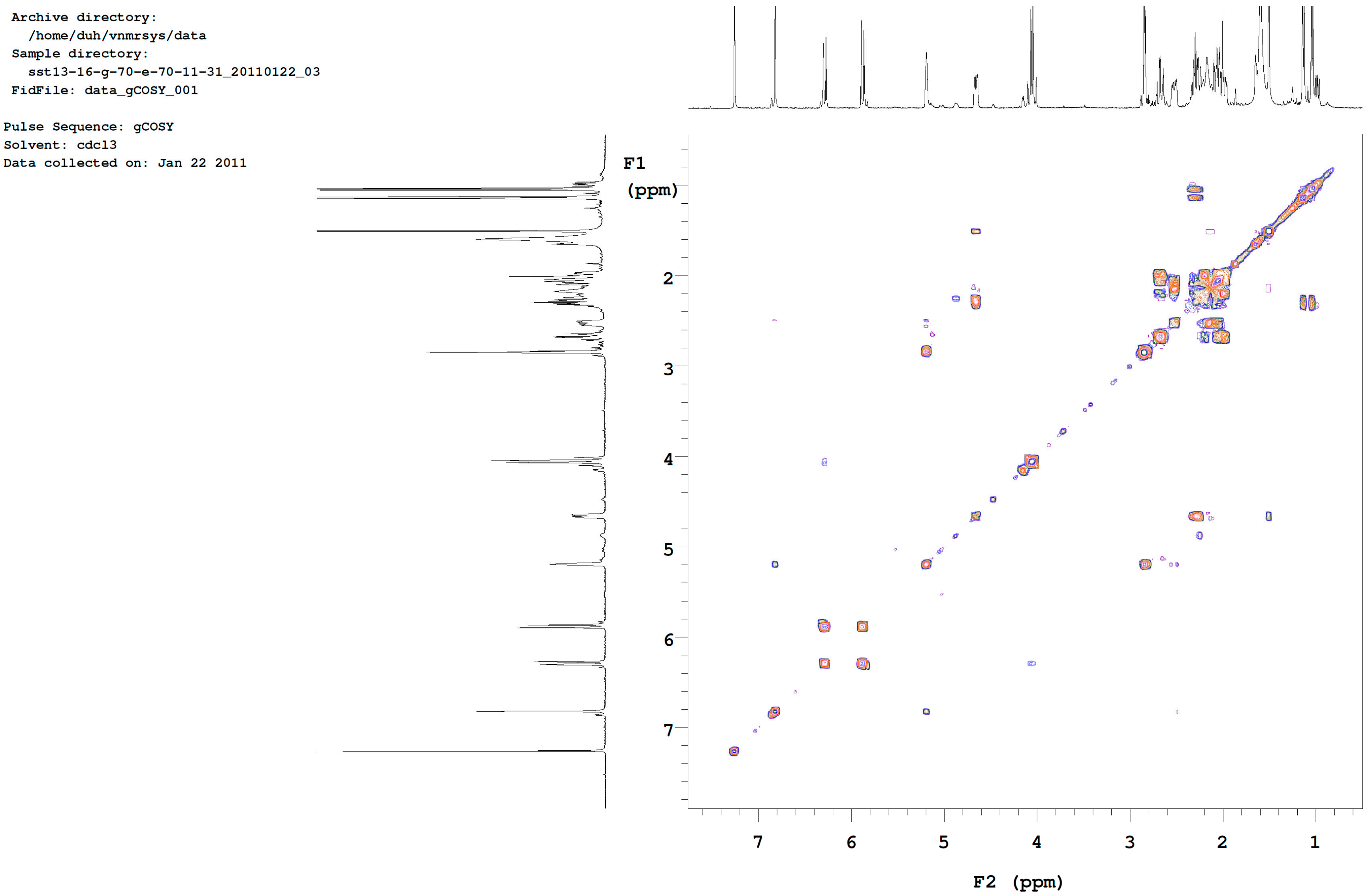

Figure S23. $^1H-^1H$ COSY spectrum (400 MHz) of ehrenbergol G (**4**) in C_6D_6.

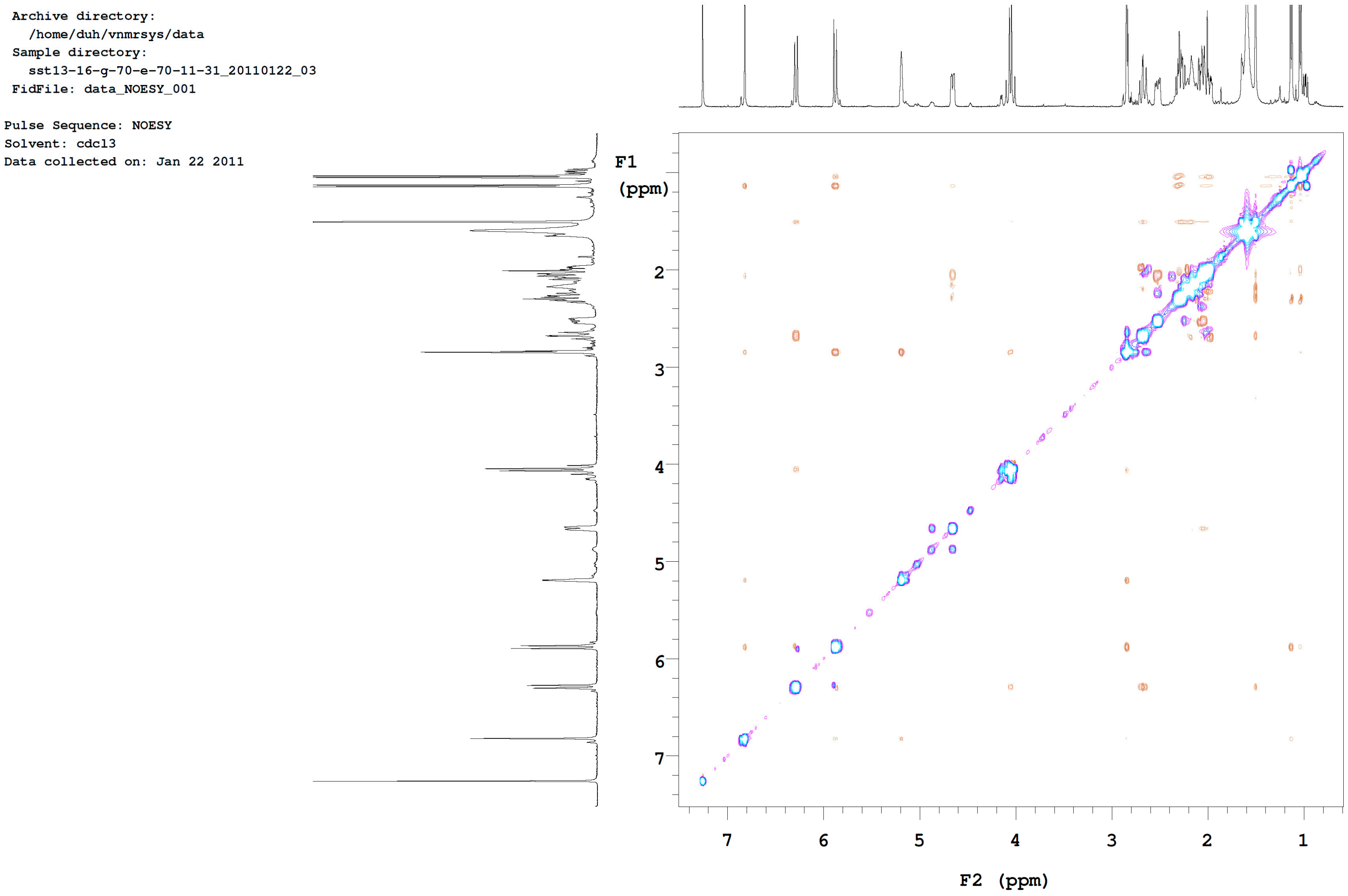

Figure S24. NOESY spectrum (400 MHz) of ehrenbergol G (**4**) in C_6D_6.

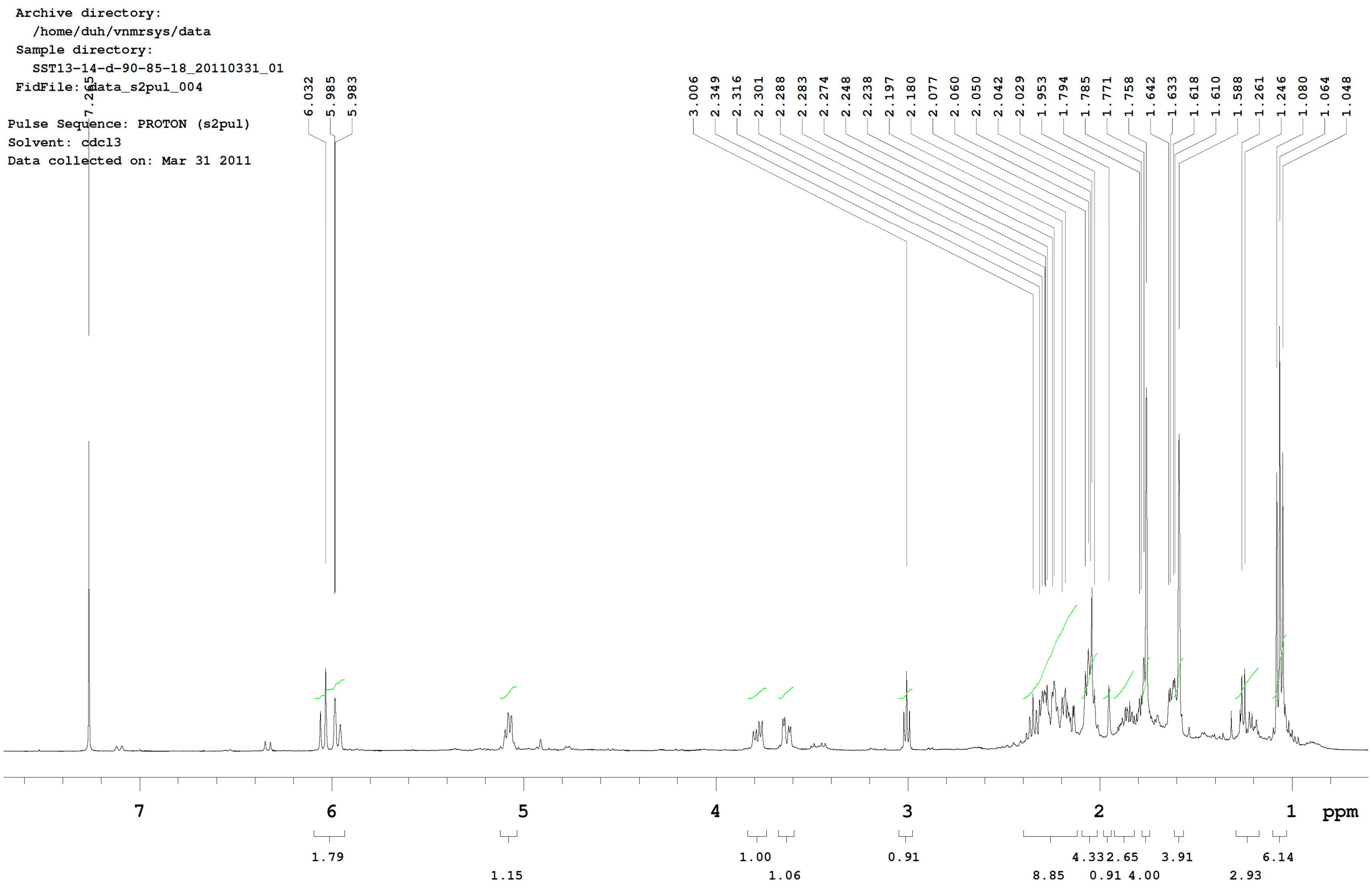

Figure S25. ^{1}H NMR spectrum (400 MHz) of ehrenbergol H (**5**) in $CDCl_3$.

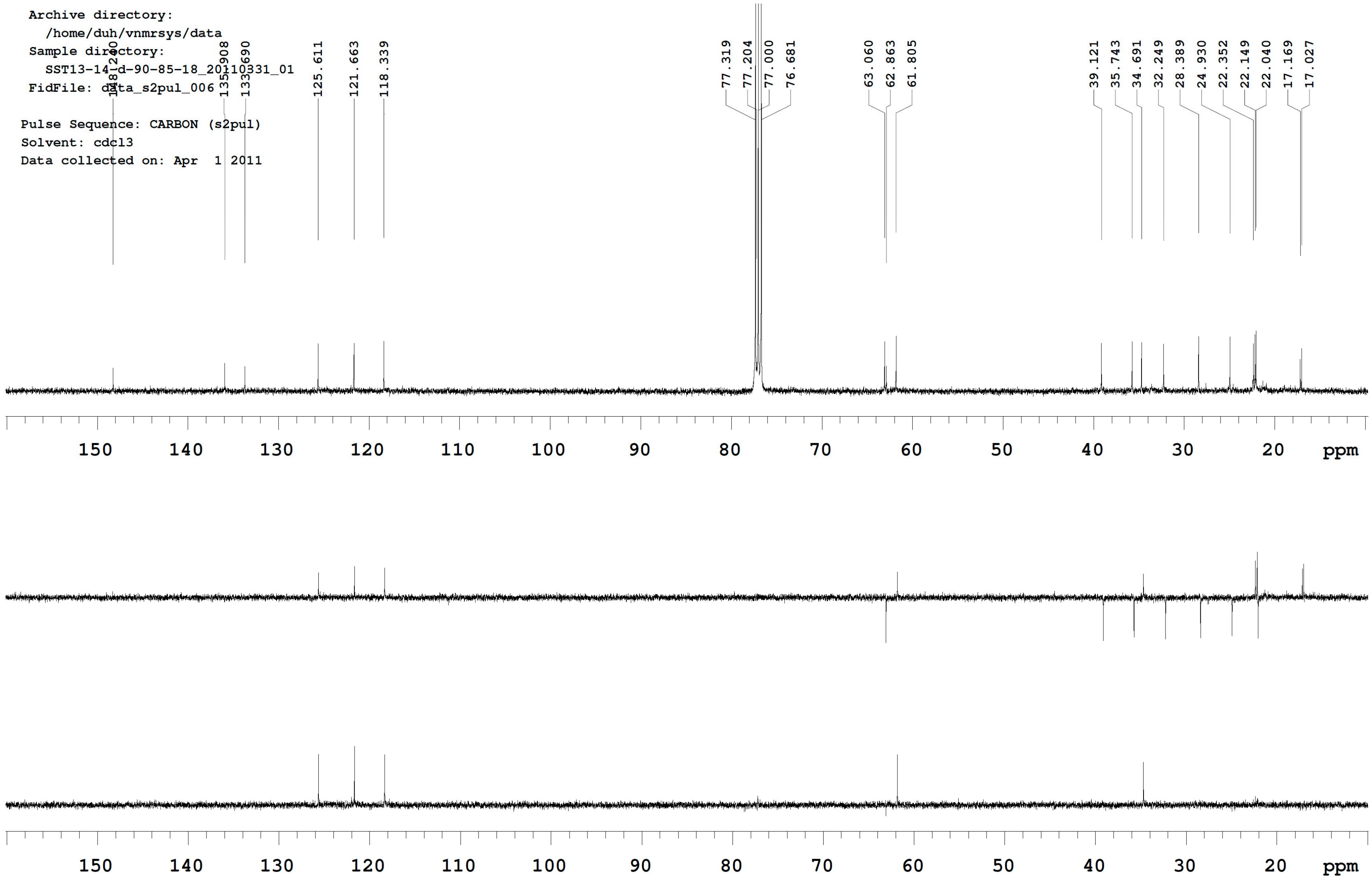

Figure S26. DEPT and ^{13}C NMR spectrum (100 MHz) of ehrenbergol H (**5**) in $CDCl_3$.

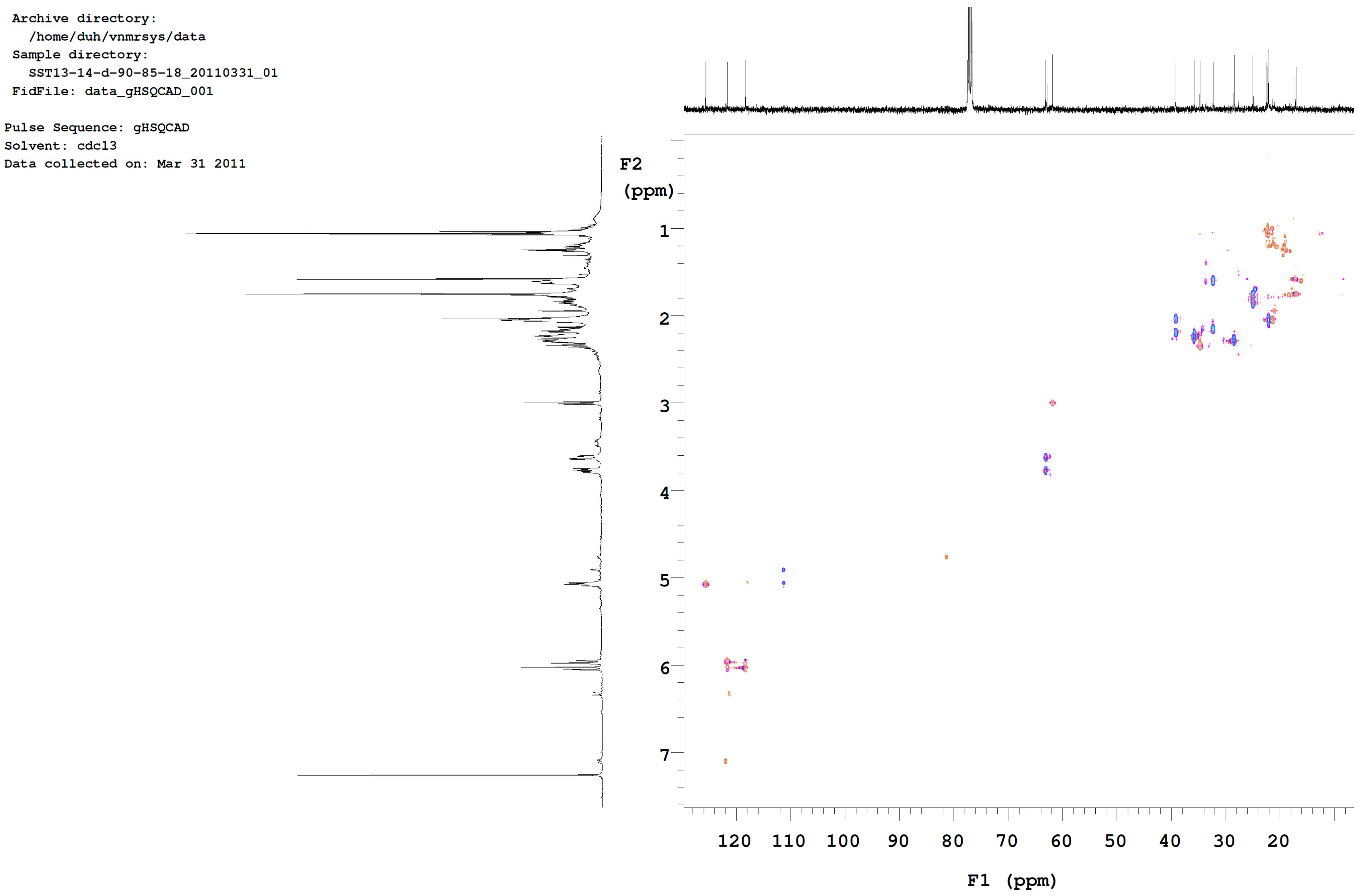

Figure S27. HSQC spectrum (400 MHz) of ehrenbergol H (**5**) in $CDCl_3$.

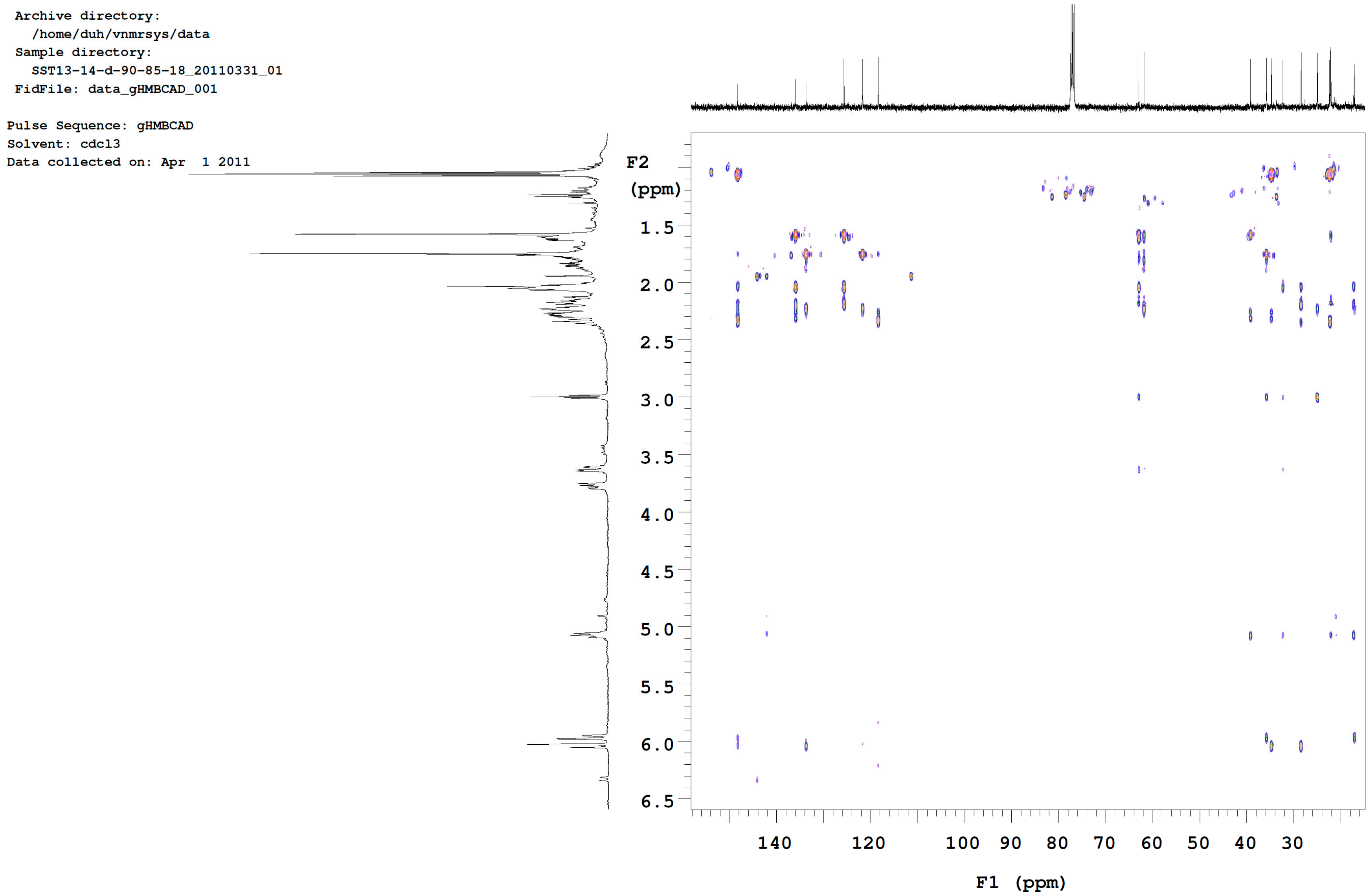

Figure S28. HMBC spectrum (400 MHz) of ehrenbergol H (**5**) in $CDCl_3$.

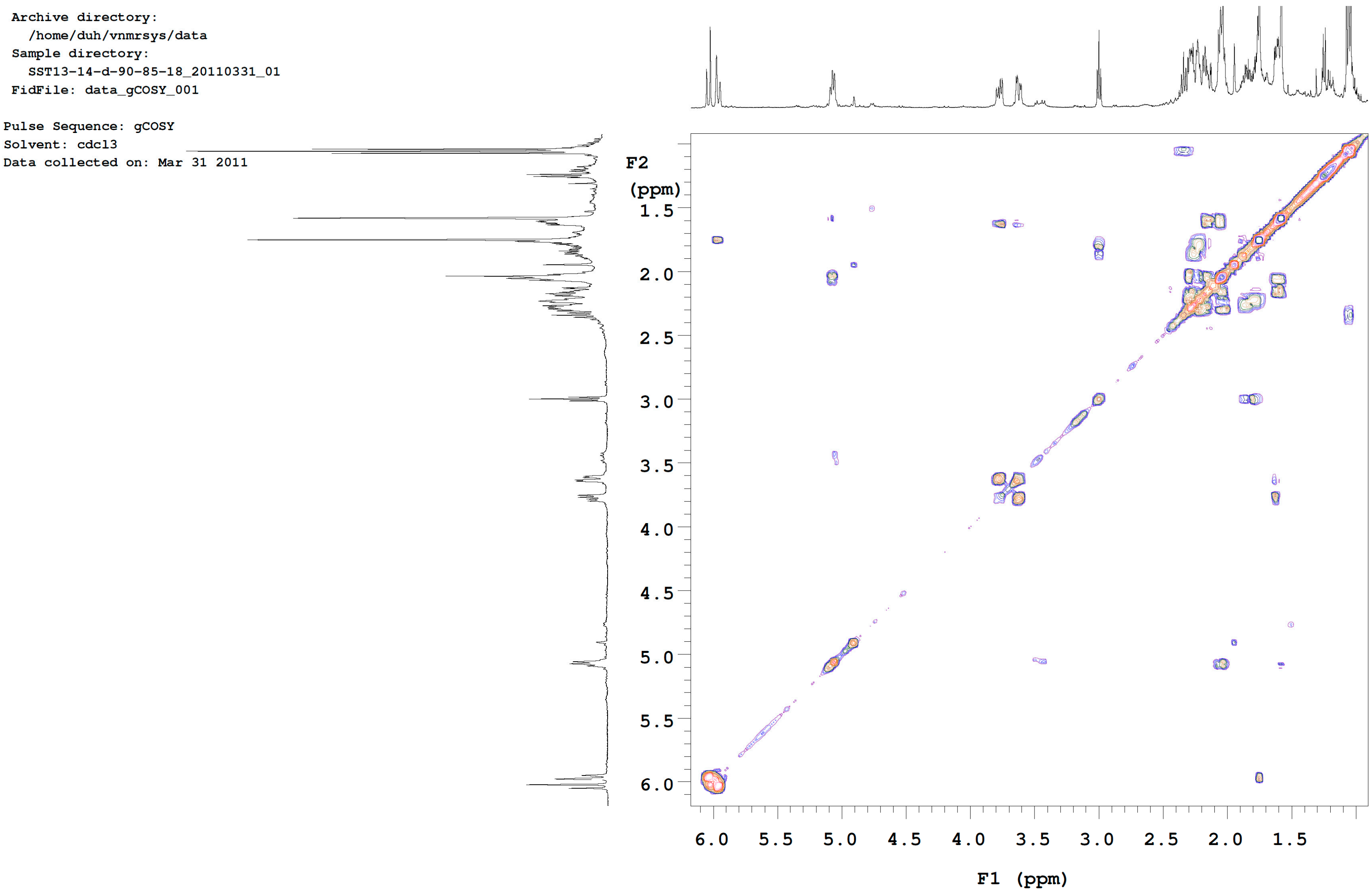

Figure S29. $^{1}H-^{1}H$ COSY spectrum (400 MHz) of ehrenbergol H (**5**) in $CDCl_3$.

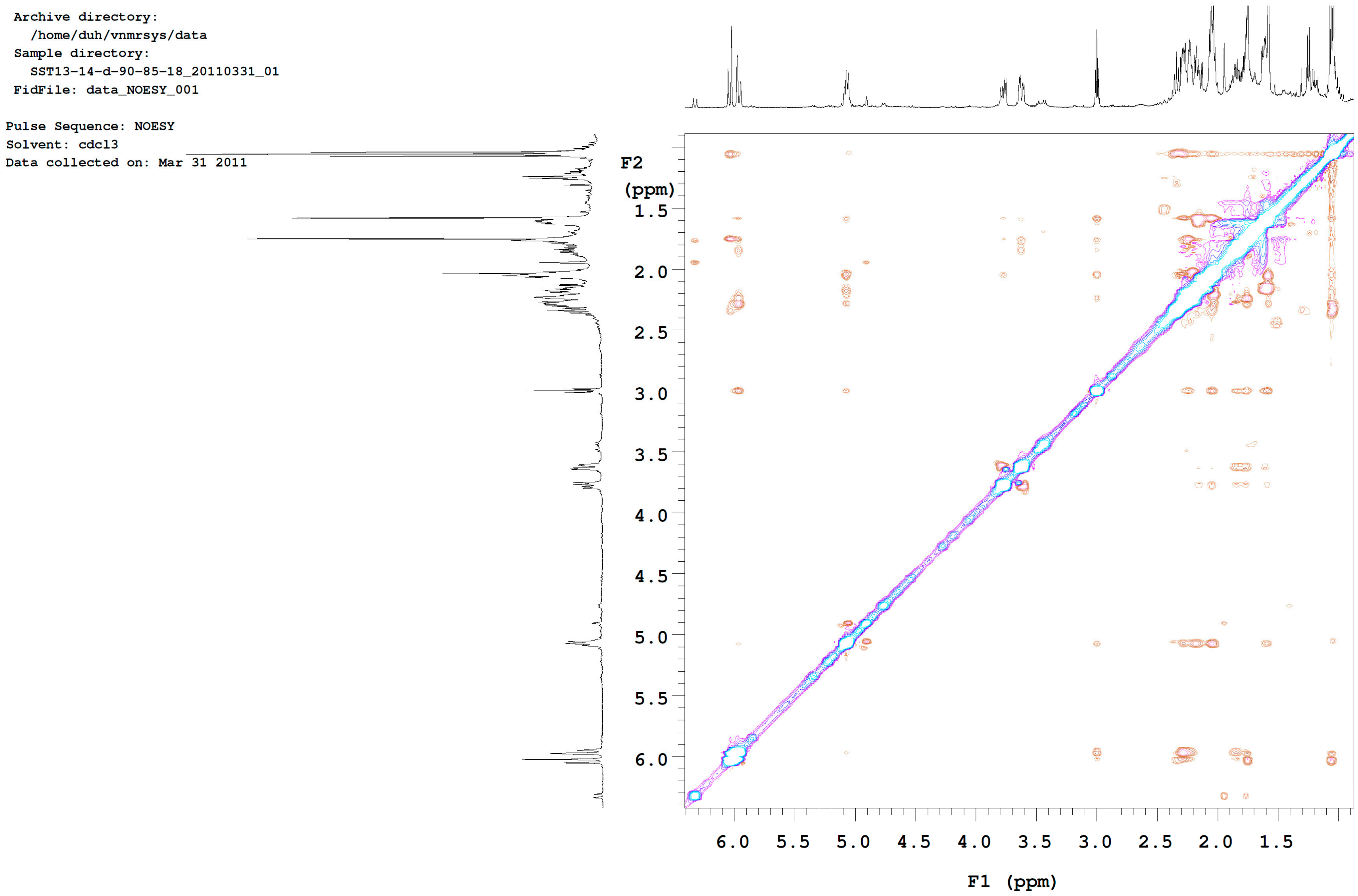

Figure S30. NOESY spectrum (400 MHz) of ehrenbergol H (**5**) in $CDCl_3$.
